# A Novel Snf2 Protein Maintains *trans*-Generational Regulatory States Established by Paramutation in Maize

**DOI:** 10.1371/journal.pbio.0050275

**Published:** 2007-10-16

**Authors:** Christopher J Hale, Jennifer L Stonaker, Stephen M Gross, Jay B Hollick

**Affiliations:** Department of Plant and Microbial Biology, University of California Berkeley, Berkeley, California, United States of America; Cold Spring Harbor Laboratory, United States of America

## Abstract

Paramutations represent heritable epigenetic alterations that cause departures from Mendelian inheritance. While the mechanism responsible is largely unknown, recent results in both mouse and maize suggest paramutations are correlated with RNA molecules capable of affecting changes in gene expression patterns. In maize, multiple *required to maintain repression (rmr)* loci stabilize these paramutant states. Here we show *rmr1* encodes a novel Snf2 protein that affects both small RNA accumulation and cytosine methylation of a proximal transposon fragment at the *Pl1-Rhoades* allele. However, these cytosine methylation differences do not define the various epigenetic states associated with paramutations. Pedigree analyses also show RMR1 does not mediate the allelic interactions that typically establish paramutations. Strikingly, our mutant analyses show that *Pl1-Rhoades* RNA transcript levels are altered independently of transcription rates, implicating a post-transcriptional level of RMR1 action. These results suggest the RNA component of maize paramutation maintains small heterochromatic-like domains that can affect, via the activity of a Snf2 protein, the stability of nascent transcripts from adjacent genes by way of a cotranscriptional repression process. These findings highlight a mechanism by which alleles of endogenous loci can acquire novel expression patterns that are meiotically transmissible.

## Author Summary

Genetics is founded on the principle that heritable changes in genes are caused by mutations and that the regulatory state of gene pairs (alleles) is passed on to progeny unchanged. An exception to this rule, paramutations—which reflect the outcome of interactions between alleles—produce changes in gene control that are stably inherited without altering the DNA sequence. It is currently thought that these allelic interactions cause structural alterations to the chromatin surrounding the gene. Recent work in both maize and mice suggests that RNA molecules may be responsible for paramutations. Several genes are required to maintain the repressed paramutant state of a maize *purple plant1 (pl1)* allele, and here we report that one of these genes encodes a protein (RMR1) with similarity to a protein previously implicated in facilitating genomic DNA modifications via small RNA molecules. Genetic and molecular experiments support a similar role for RMR1 acting at a repeated sequence found adjacent to this *pl1* gene. Although loss of these DNA modifications leads to heritable changes in gene regulation, the data indicate these changes do not represent the heritable feature responsible for paramutation. These findings highlight an unusual but dynamic role for repeated genomic features and small RNA molecules in affecting heritable genetic changes independent of the DNA template.

## Abstract 3

Paramutations are heritable epigenetic changes, which, in maize, are stabilized by multiple genes, one of which expresses a novel Snf2 protein. This protein affects the stability of transcripts from adjacent genes by a cotranscriptional repression process.

## Introduction

The term “paramutation” describes a genetic behavior in which the regulatory state of specific alleles is heritably altered through interactions with their homologous partners in *trans* [1,2]. This behavior presents an exception to the Mendelian principle that alleles segregate from a heterozygous state unchanged [3]. Paramutations have been best characterized at loci encoding transcriptional regulators of pigment biosynthesis in maize, but similar behaviors have been described in other plant and animal systems, most recently in mice [4,5]. While the broader roles of paramutation in genome-wide regulation and evolution remain to be seen, the *Pl1-Rhoades* allele of the maize *purple plant1 (pl1)* locus presents a tractable system to study the paramutation process.

The *pl1* locus encodes a Myb-like protein that acts as a transcriptional activator of genes required for anthocyanin pigment production [6]. Inheritance patterns illustrate that the *Pl1-Rhoades* allele can exist in quantitatively distinct regulatory states, reflected by differences in plant color. When individuals with a highly expressed reference state of *Pl1-Rhoades,* termed *Pl-Rh,* are crossed with plants having a repressed state, referred to as *Pl′,* only progeny with weak pigmentation are produced [7,8]. *Pl-Rh* states invariably change to *Pl*′ in *Pl-Rh/Pl′* heterozygotes [7]; this is a typical hallmark of paramutation. Relative to *Pl-Rh,* the *Pl′* state displays reductions in both *Pl1-Rhoades* RNA levels (∼10-fold) and transcription rate (∼3-fold) that are associated with a reduction in plant pigment [8]. This repressed *Pl′* state is meiotically stable when maintained in a *Pl1-Rhoades* homozygote, with no reversion to *Pl-Rh* seen to date. *Pl′* can, however, revert to *Pl-Rh* when heterozygous with some *pl1* alleles other than *Pl1*-*Rhoades,* when maintained in a hemizygous condition, or in the presence of specific recessive mutations [9–12].

Genetic screens for ethyl methanesulfonate (EMS)–induced recessive mutations identify at least ten loci, including *required to maintain repression1 (rmr1), rmr2*, *rmr6*, and *mediator of paramutation1 (mop1),* whose normal functions maintain the repressed *Pl′* state ([10,11,13]; J. B. H., unpublished data). These *rmr* mutations specifically affect the expression of *Pl1-Rhoades* and not other *pl1* alleles [10,11], indicating that the *Pl1-Rhoades* allele is a direct and specific target of paramutation-based epigenetic changes. *mop1* was recently identified [14,15] as encoding the putative ortholog of the *Arabidopsis* protein RDR2, a presumed RNA-dependent RNA polymerase involved in siRNA-based maintenance of de novo cytosine methylation [16]. Recessive mutations defining *rmr1, rmr2,* and *rmr6* destabilize the repressed *Pl′* state, resulting in darkly pigmented plant tissues, an increase in *pl1* RNA levels, and meiotic transmission of *Pl-Rh* revertant states [10,11]. To date, the molecular identity of these *rmr* factors remains unknown.

In this report we identify *rmr1* as encoding a novel Snf2 protein that represents a founding member of a subgroup of factors similar to proteins involved in plant small RNA metabolism. Our analyses show that RMR1 affects both *pl1* RNA transcript stability as well as small interfering RNA (siRNA) accumulation and DNA methylation patterns at *Pl1-Rhoades*. These results support a model in which maintenance of paramutant states is dependent on a repression mechanism similar to the recently proposed cotranscriptional gene silencing mechanism in fission yeast [17,18]. To our knowledge, RMR1 is the first protein identified that maintains *trans*-generationally repressed states established by paramutation.

## Results

### *rmr1* Defects Affect *pl1* RNA Stability

The *rmr1* locus is defined by four recessive mutations (Protocol S1) characterized by a darkly pigmented plant phenotype that results from loss of *Pl*′ repression. Previous RNase protection experiments showed a 26-fold increase in *pl1* RNA in floret tissue between *rmr1–1* mutant plants and heterozygous siblings [10]. However, these experiments did not address if changes in *pl1* transcript abundance correlated with changes in actual transcription at the *pl1* locus.

**Figure 1:**
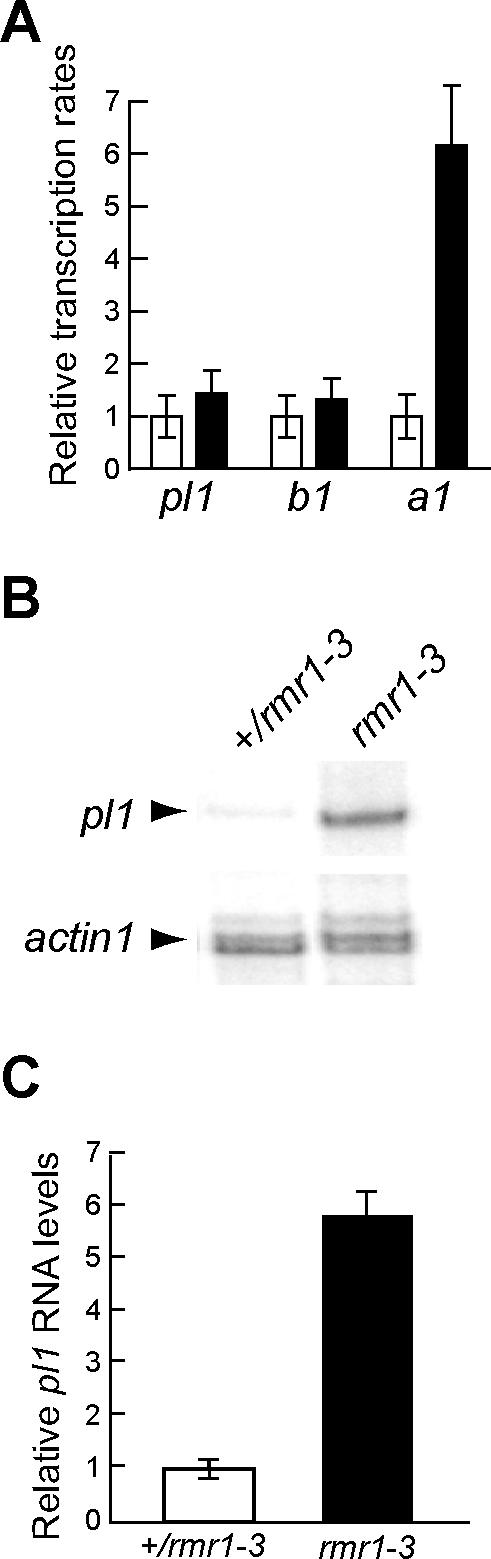
Comparison of *pl1* Expression between *rmr1* Mutants and Heterozygous Siblings *pl1* RNA levels increase significantly in *rmr1* mutants, while transcription rates of paramutant alleles are unaffected. (A) The relative mean transcription rates from four independent sets of *+/rmr1–3* (open) and *rmr1–3/rmr1–3* (filled) siblings (± standard error of the mean) at the indicated loci. (B) RNase protection analysis comparing *pl1* and *actin1* RNA levels in the same individual plants and tissues used for in vitro transcription analysis. (C) Quantification of relative *pl1* RNA levels from analyses as represented in (B).

In vitro transcription assays using nuclei isolated from husk leaf tissue revealed there was no statistically significant change in relative transcription rates of the *Pl1-Rhoades* allele between *rmr1–1* mutants and heterozygous siblings (Figure S1). However, transcription rates of *anthocyaninless1 (a1),* a direct target of the PL1 transcriptional activator [7,19], were ∼4-fold greater in *rmr1–1* mutants (Figure S1), reflecting significantly increased PL1 activity. Transcription rates from *colored plant1 (b1)*—a locus encoding a basic helix-loop-helix factor genetically required for *a1* transcription— remained unchanged. These results were recapitulated in comparisons between nuclei isolated from *rmr1–3* mutants and heterozygous siblings in which in vitro transcription assays revealed no significant change in transcription rate of *Pl1-Rhoades* (Figures 1A and S1; *n =* 4, two-tailed two-sample *t-*test, *t* = 0.8, *p* = 0.5) while RNase protection experiments showed a 5.7-fold increase in *pl1* RNA for *rmr1–3* mutants (Figure 1B and 1C; *n =* 2, two-tailed two-sample *t*-test, *t* = 10.8, *p* < 0.01) using RNA isolated from the same tissues of the same individuals. Similar comparisons from identical tissues but in a different genetic background again showed that transcription rates at *pl1* remained unchanged while *pl1* RNA levels increased 7.52-fold in *rmr1–3* mutants compared to heterozygous siblings (*n =* 1; see Protocol S1).

These RNA expression results sharply contrast those of previous reports using identical in vitro transcription assays that detected significant differences in *Pl1-Rhoades* transcription rates between *Pl′* and *Pl-Rh* states and between *rmr6* mutants and non-mutants [8,11]. This indicates our in vitro results represent an accurate assessment of transcription rates and not a limitation of the assay to detect rate differences at the *pl1* locus. Combined, these results imply an increase of *pl1* RNA abundance disproportionate to insignificant changes in transcription rate in *rmr1* mutants, the most direct interpretation being that RMR1 functions at a post-transcriptional level to stabilize *Pl1-Rhoades* RNA.

### *rmr1* Encodes a Novel Protein with a Snf2 Domain

**Figure 2:**
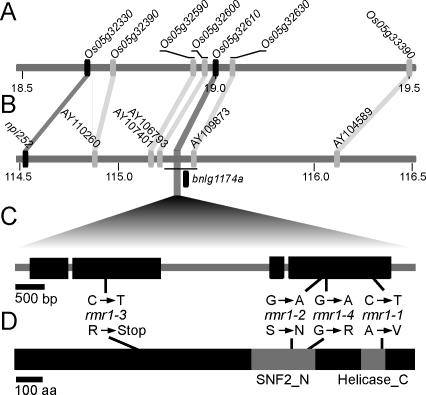
Map-Based Cloning of *rmr1* (A and B) Rice Chromosome 5 (http://rice.tigr.org/) (A) and maize Chromosome 6 (B) (2005 FPC map, contig 285; http://www.genome.arizona.edu/fpc/maize/) with synteny of annotated rice loci and orthologous maize markers (gray boxes) highlighted. Black boxes indicate the rice *rmr1* ortholog, *Os05g32610,* and the SSLP marker *npi252*. Neither *rmr1* nor SSLP marker *bnlg1174a* are represented on the FPC map, though both can be amplified from a BAC (c0007N19), identified by GenBank accession AY109873, which maps to the region identified by the black line. (C) Gene structure of *rmr1* with exons in black; EMS-derived mutations are noted. (D) Gray boxes highlight conserved Pfam SNF2_N (*E*-value = 1.3 × 10^−8^; amino acids 851 to 1214) and Helicase_C (*E*-value = 1.1 × 10^−11^; amino acids 1255 to 1334) profiles in the RMR1 protein. Predicted translational consequences of each *rmr1* mutation are indicated.

To better understand *Rmr1* function and the paramutation mechanism, we used a map-based approach to identify the *rmr1* gene. Using a polymorphic F2 population we looked for genetic linkage between the mutant phenotype and previously mapped chromosome markers [20]. The dark-color phenotype of *rmr1–1* homozygotes showed invariant cosegregation with the mutant parent polymorphism of SSLP markers *bnlg1174a* (680 chromosomes tested; <0.15 cM) and *npi252* (60 chromosomes tested; <1.7 cM), indicating *rmr1* was tightly linked to those markers in bin 6.05 on Chromosome 6. We used the high degree of synteny between this region and rice Chromosome 5 to identify candidate *rmr1* orthologs (Figure 2A and 2B).

Within the syntenic rice region we identified a gene model, *Os05g32610* (http://rice.tigr.org/), predicted to encode a Snf2 protein. The Snf2 protein family is composed of members similar to Saccharomyces cerevisiae Snf2p with a bipartite helicase domain containing Pfam SNF2_N and Helicase_C profiles, and includes many proteins involved in ATP-dependent chromatin remodeling [21,22]. While there was no public maize expressed sequence tag for this candidate, we used BLAST searches to identify genomic survey sequence similar to *Os05g32610*. Oligonucleotide primers were designed from these sequences and used to generate PCR amplicons spanning the maize *Os05g32610* ortholog, which were sequenced from individuals homozygous for *Rmr1* progenitor alleles and mutant derivatives (see Materials and Methods and Dataset S1). The maize sequence generated from each of the homozygous mutants revealed single unique transition-type base pair changes consistent with EMS mutagenesis relative to the progenitor (Figure 2C). The amino acid change associated with the *rmr1–1* allele is predicted to prevent proper folding of the helicase domain [23], while the non-conservative amino acid substitutions associated with the *rmr1–2* and *rmr1–4* alleles occur at highly conserved residues in the SNF2_N profile (Figure 2D). The *rmr1–3* allele is associated with a nonsense mutation predicted to truncate the peptide before the conserved helicase domain. CAPS markers were designed to the potential *rmr1–1* and *rmr1–3* lesions and used to show that the base pair polymorphisms at each of the probable lesions invariably cosegregate with the mutant phenotype (see Materials and Methods). These results support these polymorphisms as bona fide molecular lesions in the *rmr1* gene. Based upon molecular genetic mapping data, DNA sequencing results, and the relevance of the fact that Snf2 proteins affect chromatin environments, we conclude the *rmr1* locus encodes a protein containing a Snf2 helicase domain*.*

**Figure 3:**
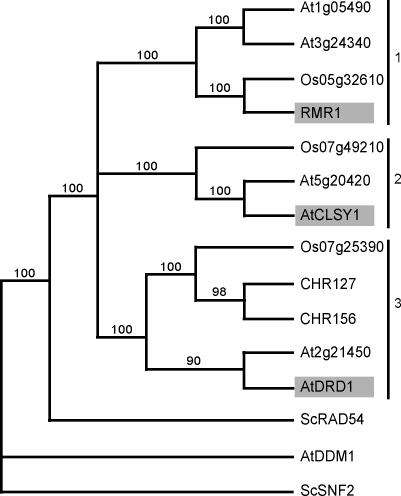
RMR1 Defines a Monophyletic Clade Distinct from DRD1 Distance tree with bootstrap values produced from alignment (Figure S2) of the predicted Snf2 domain with other Snf2 proteins: the tree shows that RMR1, CLSY1, and DRD1 (highlighted in gray) are members of a Rad54-like subfamily of Snf2 proteins. Three distinct monophyletic groups compose this subfamily, numbered 1 to 3. Prefixes: At, *Arabidopsis;* Os, rice; Sc, *S. cerevisiae.*

*Os05g32610* gene models and our cDNA sequencing analysis (see Materials and Methods) indicate *rmr1* encodes a 1,435-amino-acid protein. In addition to having the conserved Snf2 helicase domain, the protein has a large N-terminal region with no significant identity to any known or predicted proteins. Phylogenetic comparison with other known Snf2 proteins in maize, rice, *Arabidopsis,* and budding yeast shows RMR1 is a member of a Rad54-like subfamily defined by DRD1 (Figure 3). *Arabidopsis* DRD1 is a putative chromatin remodeling factor affecting RNA-directed DNA methylation (RdDM) patterns [24–26]. In the emerging RdDM pathway model, DNA sequences are targeted for de novo cytosine methylation by complementary siRNA molecules generated from “aberrant” RNA transcripts. The putative MOP1 ortholog in *Arabidopsis,* RDR2, is required in this pathway to presumably generate double-stranded RNA from these transcripts and provide a substrate for siRNA biogenesis through activity of a Dicer-like enzyme [27]. DRD1 is thought to be a downstream effector protein that facilitates de novo methylation of targeted DNA sequences, possibly by modulating chromatin architecture to provide access to de novo methyltransferases [24–26,28]. The DRD1 subfamily also includes the recently identified CLSY1 protein implicated in the systemic spreading of siRNA-mediated silencing in *Arabidopsis* [29].

Multiple sequence alignments (Figure S2) indicate RMR1 is not the structural ortholog of either DRD1 or CLSY1. The DRD1 subfamily can be divided into three distinct monophyletic groups, with RMR1, DRD1, and CLSY1 defining different groups (Figure 3). The presumed maize ortholog of DRD1 is likely one of two proteins in the DRD1 subgroup, Chromatin remodeling complex subunit R 127 (CHR127) (http://chromdb.org/), a partial protein predicted from maize expressed sequence tag sequences, or CHR156, a full-length protein predicted from maize genomic sequence (see Materials and Methods). RMR1 is more similar to *Arabidopsis* proteins predicted from *At1g05490* and *At3g24340*. RNA interference knockdowns of these putative *Arabidopsis* orthologs are known to have little to no effect in response to DNA damage [30].

Taking into account the phylogenetic analysis of the predicted coding sequence, it is possible RMR1 function may be similar to, but distinct from, that of DRD1 and CLSY1. The three proteins may fulfill a similar role in RdDM, but perhaps function under different conditions or in distinct genomic contexts. Alternatively, they could perform different roles within an RdDM pathway, or function in separate epigenetic mechanisms altogether. Given the results of our *pl1* RNA expression analyses, it is possible that RMR1 represents a Snf2 protein that links chromatin organization to RNA transcript stability.

### RMR1 Maintains Cytosine Methylation and Small RNA Accumulation at *Pl1-Rhoades*

In the described *Arabidopsis* RdDM pathway, DRD1 maintains cytosine methylation at nonsymmetrical CNN sequences represented by siRNAs [24–26]. Many endogenous genomic targets of DRD1 appear to be repetitive elements [31]. At *Pl1-Rhoades* there is a 402-bp terminal fragment of a CACTA-like type II DNA transposon, similar to *doppia,* 129 bp upstream of the translational start site [8,32,33]. Assuming analogous functional roles of RMR1 and DRD1 we compared DNA methylation patterns at this upstream repetitive element in *rmr1* mutants and non-mutant siblings.

**Figure 4:**
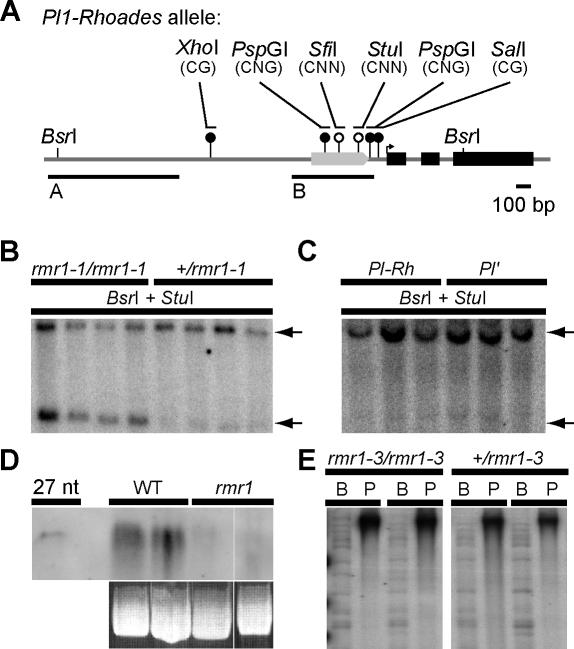
Cytosine Methylation Patterns and Small RNA Accumulation Are Altered at *Pl1-Rhoades* in *rmr1* Mutants (A) Schematic of *Pl1-Rhoades* locus with exons highlighted in black and the upstream *doppia* element represented by the gray arrow. The methylation context of sites cut by methylation-sensitive enzymes are shown in parentheses. Open circles denote sites hypomethylated in *rmr1–1* mutants while filled circles are sites methylated in both wild-type and *rmr1* mutants. BsrI restriction sites and the regions used to generate probes for blot hybridization analysis, denoted A and B, are also shown. (B and C) Representative Southern blots hybridized with probe A showing methylation status at a StuI site in *rmr1* mutants and heterozygous siblings (B), as well as *Pl′* and *Pl-Rh* plants (C) with a larger 2.9-kb band (upper arrow) representative of a fully methylated BsrI fragment, and a 2.1-kb band (lower arrow) indicative of a hypomethylated StuI site. Additional primary blots shown in Figures S3 and S8. (D) Small RNA northern blot probed with *doppia* sequence from probe B showing changes in amount of small RNAs between *rmr1–1* plants and wild-type (WT) siblings. (E) Southern blot of genomic DNA digested with BstNI (“B” lanes) and methylation-sensitive PspGI (“P” lanes) hybridized with probe B, showing no bulk changes in *doppia* methylation genome-wide.

Previous restriction-enzyme-based comparisons of DNA methylation status between *Pl-Rh* and *Pl′* states found no differences, although few 5′ proximal sites were evaluated [8]. Using Southern blot hybridization analysis following digestion of genomic DNA with methylation-sensitive restriction enzymes, we found that the *doppia* fragment is hypomethylated at specific sites in plants homozygous for the *rmr1–1* mutation compared to heterozygous wild-type siblings (Figures 4A, 4B, and S3). Consistent with findings in *Arabidopsis* RdDM mutants [16,34–36], the sites hypomethylated in *rmr1* mutants were of the CNN context. A relative hypomethylation pattern in 5′ sequences is also present in plants homozygous for mutations at either *rmr6* or *mop1* (Figures S4 and S5). In *rmr6* mutants the extent of hypomethylation was greater than that of either *rmr1* or *mop1* mutants and encompassed CG methylation sites as well as non-CG targets, suggesting *Rmr6* has a broader effect in cytosine methylation maintenance. The presence of these methylation differences in multiple mutant backgrounds indicates that this hypomethylation pattern reflects the chromatin status at *doppia* in plants where maintenance of repressed paramutant states is compromised.

Consistent with the *Arabidopsis* RdDM model, small RNAs (∼26 nt) with sequence similarity to the *doppia* element are detected in wild-type *Pl′* plants in both sense and antisense orientations (Figures 4D and S6). These small RNAs are undetectable in *rmr1* mutants, unlike in wild-type siblings. This result contrasts those in *Arabidopsis* showing that *DRD1* deficiencies do not affect the abundance of endogenous siRNAs representing repetitive elements [31]. However, it has been reported that the abundance of endogenous siRNA and *trans*-acting siRNA populations are highly reduced in *CLSY1* mutants [29].

To test if the *doppia* fragment hypomethylation was indicative of genome-wide changes we assayed the cytosine methylation status at centromeres and 45S repeat sequences. Cytosine methylation patterns were unaffected in either of these regions in *rmr1* mutants as compared to non-mutant siblings (Figure S7). Additionally, we examined the methylation status of *doppia*-like loci genome-wide (Figure 4E) and found no obvious differences between *rmr1* mutants and non-mutant siblings. These results indicate that while RMR1 acts on the *doppia* sequence upstream of *Pl1-Rhoades, doppia* elements appear unaffected throughout the genome. This specificity of RMR1 function may be due to its intimate and exclusive involvement with alleles that undergo paramutation, or may be indicative of differential regulation of repetitive elements depending on their genomic and epigenetic context.

If RMR1 is involved in maintaining cytosine methylation patterns characteristic of repressed paramutant states then a prediction would be that the methylation differences seen between mutants and non-mutants would reflect the *Pl′* and *Pl-Rh* regulatory states. Surprisingly, there are no methylation differences at the *doppia* fragment between *Pl-Rh* and *Pl′* states (Figures 4C and S8). These results suggest that while the upstream *doppia* element of *Pl1-Rhoades* is a target of multiple factors involved in maintaining the epigenetic repression associated with paramutation, the actual process of paramutation does not result in similar changes of DNA methylation at this element.

### RMR1 Is Not Required for Establishment of Paramutant States

**Figure 5:**
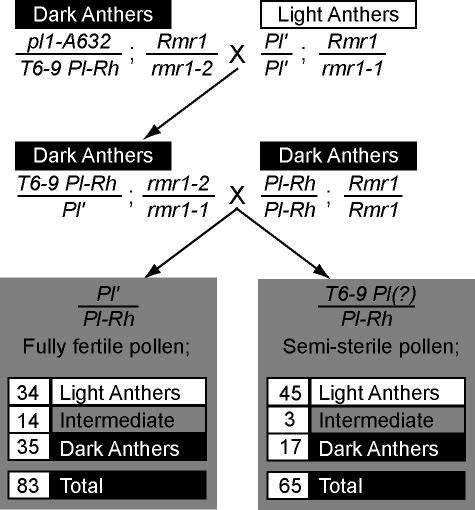
*Pl′* Establishment in an *rmr1* Mutant Background Plants with a T6–9 translocation chromosome carrying *Pl1-Rhoades* in the *Pl-Rh* state (dark anther pigmentation) and heterozygous for the *rmr1–2* allele were crossed to *Pl′* plants (light anther pigmentation) heterozygous for the *rmr1–1* allele. Of the resultant progeny with semi-sterile pollen (heterozygous for the T6–9 interchange pair), plants homozygous for a mutation at *rmr1* were chosen based on the dark anther phenotype. These plants were then crossed to a *Pl-Rh* tester with the expectation that in progeny inheriting the interchange, the expression status of the *Pl1-Rhoades* allele on the T6–9 translocation chromosome (*T6–9 Pl(?)*) would indicate if establishment of the *Pl′* state was affected in the F1. The numbers represent the number of plants displaying a given anther phenotype, indicating that the *Pl′* state was established on the interchange chromosome in the *rmr1* mutants.

Based on a reverse transcriptase PCR (RT-PCR) expression profile (Figure S9) *rmr1* appears to be expressed in all rapidly dividing somatic tissues, consistent with a role in maintaining paramutant states throughout development. However, since the methylation patterns maintained by RMR1 appear unrelated to the paramutant state of *Pl1-Rhoades,* we questioned whether RMR1 is directly required for paramutation to occur. This process results in the invariable establishment of the *Pl′* state in *Pl′/Pl-Rh* plants, as evidenced by the observation that only *Pl′/Pl′* progeny are found when *Pl′/Pl-Rh* plants are crossed to *Pl-Rh/Pl-Rh* testers [7,8]. If RMR1 were directly involved in this process we would expect that an *rmr1* deficiency might interfere with the *Pl′* establishment event. To test this, we tracked the behavior of individual *Pl1-Rhoades* alleles in test crosses to assess the ability of the *Pl′* state to facilitate paramutations in *Pl′*/*Pl-Rh; rmr1–1*/*rmr1–2* plants. The *Pl1-Rhoades* allele in a *Pl-Rh* state was genetically linked (∼1.5 cM) to a T6–9 translocation breakpoint *(T6–9)*. The T6–9 interchange can act as a dominant semi-sterility marker, allowing us to trace specific *Pl1-Rhoades* alleles through genetic crosses [11]. *rmr1* mutants heterozygous for the T6–9 interchange *(T6–9 Pl-Rh/Pl′)* were crossed to a *Pl-Rh/Pl-Rh* tester (Figure 5; Table S1). If establishment of the *Pl′* state was prevented in *rmr1* mutants, we would expect all progeny receiving the interchange to display a *Pl-Rh*/*Pl-Rh* phenotype (dark anther pigmentation). We observed that over half the progeny inheriting the interchange displayed a *Pl′/Pl′-*like phenotype (light anther pigmentation), indicating that paramutation was established in the *rmr1* mutant parent. It should also be noted that *Pl*-*Rh*/*Pl-Rh* plants, and those of an intermediate phenotype of partial pigmentation [7], were present in both progeny inheriting the interchange and those inheriting a normal chromosome. These results are consistent with previous work showing *Pl′* can revert to a *Pl-Rh* state in *rmr1* mutants [10].

Corresponding analysis of the establishment of paramutant states at the *b1* locus generated similar results (Table S2). The repressed *B′* state of the *B1-Intense* allele [37] was established in *B′*/*B-I rmr1* mutants greater than 95% of the time. While it is possible that *rmr1* defects affect establishment efficiency, it will be difficult to differentiate any such effects from its clear role in maintenance [11]. These results point to an interesting duality in RMR1 function in which the wild-type protein is necessary for meiotic heritability of repressed epigenetic states, but is not required to establish these states. This duality is markedly different from results generated in the analysis of *DRD1,* which was shown to be necessary for the maintenance, establishment, and removal of repressive epigenetic marks [24,25].

## Discussion

RMR1 is the first protein identified whose function acts to maintain *trans*-generationally repressed states associated with paramutation, a genetic behavior that affects meiotically heritable epigenetic variation through allelic interactions at endogenous loci. The identification of RMR1 as a Snf2 protein highlights an emerging role of these proteins in establishing and maintaining epigenetic marks. In *Arabidopsis* the Snf2 proteins DRD1 and DDM1 [38,39] are known to maintain cytosine methylation patterns. Lsh1, the mammalian protein most closely related to DDM1, is also required for normal DNA methylation patterns [40–42]. There are some 42 Snf2 proteins in *Arabidopsis* and at least as many in maize (http://chromdb.org/). This diversity likely represents great functional specialization amongst these proteins. We have placed RMR1 in an RdDM pathway based on its helicase domain similarity to DRD1 and the recent identification of MOP1 as an RDR2 ortholog [14,15]. Consistent with this proposed pathway, the *rmr1* mRNA expression profile (Figure S9) closely matches that of *mop1* [15]. Additionally, both RMR1 and MOP1 are necessary to maintain cytosine methylation patterns at silenced transgenes [43], the *Pl1-Rhoades doppia* sequences, and certain *Mutator* transposable elements ([15,44]; J. B. H. and D. Lisch, unpublished data). DRD1 is also known to target repetitive elements found in euchromatic contexts through an RdDM pathway [31]. However, the role RMR1 plays to maintain the repressed paramutant states at *Pl1-Rhoades* appears different than the function of DRD1 in the *Arabidopsis* RdDM pathway, as RMR1 has, in addition to its requirement for CNN methylation at *doppia,* a role in the normal accumulation of small RNAs with similarity to that element.

It is unclear how RMR1 mediates the post-transcriptional regulation of *pl1* transcripts as suggested by the in vitro transcription and RNase protection assays reported here. It is possible that *pl1* transcripts resulting from *Pl1-Rhoades* in the *Pl′* state are less stable than those produced from the *Pl-Rh* state because of differences in the chromatin environment of *Pl1-Rhoades.* However, there do not appear to be any *Pl′*-specific small RNAs produced from the *pl1* coding region [12]. In S. pombe it has been shown that the chromatin environment of a locus can affect RNA transcript levels without altering RNA polymerase II occupancy of that locus, leading to the proposal of a cotranscriptional gene silencing mechanism whereby nascent transcripts initiating in a heterochromatic environment are degraded by complexes targeted via heterochromatic small RNAs [17,18]. Chromatin differences in the upstream region of *Pl1-Rhoades* may favor recruitment of alternative RNA-processing factors or RNA polymerases, which in turn influence the stability of *pl1* transcripts. In plants, localization of the large subunit 1a of RNA polymerase IV to loci targeted for RdDM appears necessary for the biogenesis of siRNAs from these loci [28]. When *Pl′* repression is disrupted in *rmr1* mutants, this alternate genesis or processing of the *pl1* transcript may also be lost. Alternatively, our results may highlight a novel role for RMR1-like Snf2 proteins in directly interacting with nascent RNA transcripts via a helicase domain, or in recruiting factors that directly destabilize these transcripts.

**Figure 6:**
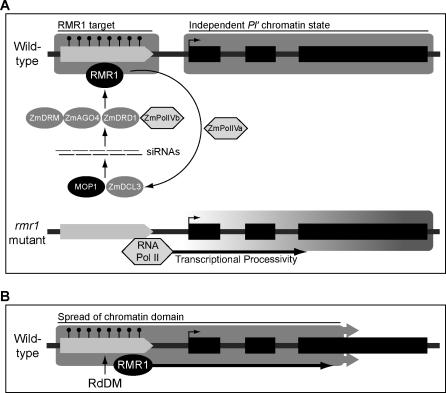
Two General Models for RMR1 Action at the *Pl1-Rhoades* Allele RMR1 maintains nonsymmetrical methylation of the *doppia* element (light gray arrow) upstream of the *pl1* coding region (exons in black) via an RdDM pathway. Small RNAs are produced in a RMR1-dependent fashion with homology to the *doppia* element, and maize orthologs of characterized RdDM proteins, as well as RMR1, then act as effectors of these siRNAs, facilitating cytosine methylation at complementary sequences of the DNA template. In the model shown in (A), the heterochromatic region of *doppia* is maintained and established independently of the *Pl1-Rhoades* chromatin state, but derepression of the upstream repetitive element in an *rmr1* mutant causes changes in the nearby genic region through processivity of RNA polymerase II or other general transcription factors that bind the upstream elements. In the model shown in (B), the *doppia* element is repressed by the same RdDM pathway shown in (A), but the *Pl*′ state represents a spread of the heterochromatic domain beyond the region targeted by the siRNAs for cytosine methylation. This spread might be mediated by RMR1 activity, or by another chromatin modifier. In (B), loss of RMR1 would lead to a loss of the repressive chromatin state at *doppia* and the ability for it to spread.

Importantly, our analysis of *rmr1* mutants calls into question the relationship between RMR1 function and the mechanism of paramutation at *Pl1-Rhoades.* The mutational screens identifying *rmr1, rmr6,* and *mop1* were designed to discover genetic components necessary to maintain the repressed state of *Pl′*, not necessarily factors needed to establish this repressed state [10,13]. Therefore, it is possible that loci thus far identified may be indirectly related to the paramutation mechanism. Our results are consistent with a model wherein RMR1 functions in an RdDM pathway, along with an RDR2-like enzyme, MOP1, to maintain a persistent heterochromatic-like chromatin structure at the repetitive element found directly upstream of the *pl1* coding region. While it is not clear where RMR1 acts in this pathway it presumably acts coordinately with the maize orthologs of known RdDM components identified in *Arabidopsis,* namely DCL3 [16,45], the DRM methyltransferases [36], AGO4 [46,47], the RNA polymerase IV subunits, and the maize DRD1 ortholog (Figure 6A). In this model, *doppia* transcripts, perhaps because of the repetitive nature of the *doppia* genomic elements and/or the numerous internal subterminal repeats that are present in these elements [32,48], are the source of aberrant RNA that is processed via MOP1 and a DCL3 enzyme into siRNAs. This small RNA production is carried out in a manner that is dependent on RMR1 activity, possibly via direct interaction with a small RNA processing complex or by making the DNA accessible to factors necessary for siRNA precursor generation such as polymerase IVa. These siRNAs, through the activity of AGO4, DRM enzymes, and polymerase IVb, then establish a heterochromatic state at the *Pl1-Rhoades doppia*-like element that is present in both *Pl-Rh* and *Pl′* states. The methylation effects seen in *rmr1* mutants might indicate that this heterochromatization machinery depends on the activity of RMR1 to feed back on the *doppia* element, or loss of RMR1 may short circuit this pathway and thus affect methylation activity indirectly. An RMR1 defect then affects stability of paramutant states at *pl1* because of the chromatin context of the *Pl1-Rhoades* allele, and not through direct disruption of components required for paramutations to occur. This is in line with a report that MOP1-dependent small RNAs produced at the *b1* locus are insufficient to mediate paramutation [49].

The relationship between RMR1 action, the chromatin organization of *Pl1*-*Rhoades,* and the repressed *Pl′* state is not clearly understood at this time. It is possible that derepression of the upstream repetitive element makes the region more accessible to general transcription factors whose actions could destabilize repressive *Pl′* chromatin states that are independent of those maintained at *doppia* (Figure 6A). Indeed, RNA polymerase processivity can lead to changes in the chromatin environment through histone modifications or histone replacement [50,51]. Alternatively, *Pl′* chromatin states may represent a spreading of the heterochromatic domain at *doppia* into a euchromatic region defined by the *Pl1-Rhoades* gene space (Figure 6B). In fission yeast, heterochromatic domains nucleated by small RNAs have the ability to spread in *cis* through successive H3 K9 methylation [52]. In this situation, loss of RMR1 function would alleviate *Pl′* repression by disrupting maintenance of this expanded heterochromatic domain. In either of these situations RMR1 affects *Pl1-Rhoades* paramutations by virtue of its role in maintaining heterochromatic states at a proximal repetitive element.

McClintock was the first to describe derivative alleles in which transposons acted to control the expression patterns of attendant genes [53]. It is now clear that epigenetic modulations of the transposons themselves—what McClintock referred to as “changes in state”—can alter the regulatory properties of individual genes both somatically [54] and *trans*-generationally [55,56]. Our results indicate that even transient changes in state of the *Pl1-Rhoades doppia* fragment can have *trans*-generational effects on *pl1* gene expression patterns. These experimental examples, in the context of McClintock's thesis [53], point to a dynamic source of regulatory, and potentially adaptive, variation adjunct to the DNA itself. Precisely how this epi-variation relates to existing genome structure and function, as well as its evolutionary potential, remains a largely unexplored area of investigation.

Currently, well-characterized examples of paramutation are limited to loci where expression states have a clear phenotypic read-out, such as pigment synthesis. *cis*-Elements required to facilitate paramutation have been functionally identified at specific alleles of *b1* and *colored1 (r1)* [57–59]. To date, there is no evidence that the chromatin status of these *cis*-elements is affected by mutations at *trans*-acting loci required for maintenance of repressed paramutant states. It appears that paramutations represent a type of emergent system wherein genomic context and maintenance of chromatin states interact to facilitate meiotically heritable epigenetic variation. In this view, it is possible that *cis-* and *trans*-elements necessary for maintenance of such variation might not interact in a direct and predictable manner. What remains to be seen is the extent to which this type of system acts throughout the genome. Genome-wide screens for paramutation-like behavior, in which expression states are affected by allele history, remain technologically and conceptually challenging. Recent work by Kasschau et al. [60] suggests that in *Arabidopsis,* few endogenous genes are regulated by proximal presumed RdDM targets. However, it is tempting to speculate that examples of paramutation represent an exception to this trend, representing a mechanism by which populations can quickly, and heritably, change their transcriptome profile and regulation.

## Materials and Methods

### Scoring of the *Pl1-Rhoades* allele expression state and *rmr* mutants.

Plants were scored as carrying *Pl-Rh* or *Pl′* states through visual inspection of anther pigmentation and assignment of an anther color score as previously described [7]. *Pl′*/*Pl′* (anther color score 1 to 4) anthers show little to no pigmentation while *Pl-Rh*/*Pl-Rh* (anther color score 7) anthers are dark red to purple. Mutants were scored in the same way, with *rmr* and *mop* mutants showing a *Pl-Rh*/*Pl-Rh*-like phenotype, except in the case of the F2 *rmr1* mapping populations, in which mutants were chosen on the basis of a dark seedling leaf phenotype [10].

### Genetic stocks*.*

Elite inbred lines (B73, A619, and A632) were provided by the North Central Regional Plant Introduction Station (http://www.ars.usda.gov/main/site_main.htm?modecode=36-25-12–00). Color-converted versions of A619 and A632 inbred lines were created by introgressing the *Pl1-Rhoades* allele into each [11]. The *rmr1–1, rmr1–2, mop1–1,* and *rmr6–1* alleles have been previously described [8,10,13]. The *rmr1–3* allele was derived from identical materials used to isolate *rmr1–1* and *rmr1–2; rmr1–4* was derived from EMS-treated pollen from an A619 color-converted line applied to a color-converted A632 line [11] (see Protocol S1 and Table S3 for complementation tests). The T6–9 translocation line carrying the *Pl1-Rhoades* allele used in *Pl′* establishment tests has been described previously [11].

### *pl1* expression analyses*.*

In vitro transcription assays (*rmr1–1* and *rmr1–3;* Figures 1 and S1) and RNase protection assays (*rmr1–3* only; Figure 1) were carried out as described [8] with husk nuclei and RNA isolated from single ears of the same genetic stocks used to measure *pl1* RNA differences in *rmr1–1* anthers [10]. The *b1* and *pl1* genotypes of these plants are as follows: *B1-Intense* (*B-I*)/*B-I; Pl1-Rhoades (Pl′) Rmr1*/*Pl′ rmr1–1* and *B-I*/*B-I; Pl′ rmr1–1*/*Pl′ rmr1–1,* or *B-I*/*B-I; Pl′ Rmr1*/*Pl′ rmr1–3* and *B-I*/*B-I; Pl′ rmr1–3*/*Pl′ rmr1–3.* Identical procedures were applied to single ears from plants homozygous for *Pl′* and either homozygous or heterozygous for *rmr1–3* following a single backcross into the KYS inbred line [12]. Additional details regarding stock syntheses are available upon request.

### Genetic mapping of *rmr1*.

A F2 mapping population was created from inbred (S9) *rmr1–1/rmr1–1, Pl′/Pl′,* and color-converted A632 inbred (*Pl′/Pl′*, >93% A632) parents. DNA was isolated using the DNeasy 96 plant kit (Qiagen, http://www1.qiagen.com/) from F2 mutant seedlings, mapping parents, and F1 hybrid leaf tissue. These DNA samples were screened with SSLP markers developed from the Maize Mapping Project (http://www.maizemap.org/; US National Science Foundation award number 9872655; primer sequences and protocol available at http://maizegdb.org/). Initial marker choice was restricted to Chromosomes 6 and 9 because of linkage of *rmr1* to a T6–9 breakpoint. In addition to the *rmr1–1* mapping population, a second F2 mapping population created with inbred (S7) *rmr1–3/rmr1–3*, *Pl′/Pl′,* and color-converted A632 parents showed similar cosegregation with marker *bnlg1174a* (178 chromosomes tested; <0.56 cM). CAPS [61] markers were designed to test cosegregation of the *rmr1–1-* and *rmr1–3*-associated lesions with the *rmr1* mutant phenotype (see Protocol S1 for details). No recombinant chromosomes (876 chromosomes tested for *rmr1–1,* 268 chromosomes tested for *rmr1–3*) were found using either marker.

### Candidate gene selection and sequencing.

A BLAST search using the rice *Os05g32610* ORF as a query identified maize GSS and sorghum expressed sequence tag sequences that were used to generate a contig representing the putative maize gene (see Protocol S1 for sequence identifiers). Oligonucleotide primers (Sigma-Genosys, http://www.sigmaaldrich.com/Brands/Sigma_Genosys.html) were designed from these sequences and used in PCR amplification of genomic DNA from three separate individuals homozygous for each *rmr1* mutant allele as well as functional reference alleles *Rmr1-*B73, *Rmr1*-A632, and *Rmr1-*A619. PCR amplicons were purified using QIAquick gel extraction kit (Qiagen) and dideoxy sequenced (UC Berkeley DNA Sequencing Facility, http://mcb.berkeley.edu/barker/dnaseq/). To verify the intron/exon structure of *rmr1,* cDNA was generated from *rmr1–1* mutants as well as non-mutant B73 plants as described [15], and *rmr1* was amplified via RT-PCR. The resulting products, which were the predicted size for spliced *rmr1* transcript, were sequenced to validate the intron/exon structure shown in Figure 2C. See Protocol S1 and Table S4 for all oligonucleotide primer sequences used.

### Phylogenetic analysis.

Sequencing reads from genomic and cDNA were aligned and edited with Sequencher (Gene Codes, http://www.genecodes.com/) to create a contig representing *rmr1*. The N-terminal prediction is based on alignment of RMR1 with the protein model for *Os05g32610*. A search of the Pfam database (http://www.sanger.ac.uk/Software/Pfam/) with the predicted RMR1 protein sequence was used to identify the conserved SNF2_N and Helicase_C protein profiles of the Snf2 helicase domain. MUSCLE [62] was used to generate an alignment between RMR1 and proteins from *Arabidopsis,* rice, maize (CHR127 and CHR156), and budding yeast over the helicase domain (Figure S2). Sequences for CHR127 and CHR156 were retrieved from ChromDB (http://www.chromdb.org/). Additional sequence information for CHR156 was identified from BAC CH201-3L17 (GenBank accession AC194602), and gene model prediction was performed using FGENESH+ (Softberry, http://www.softberry.com/) with RMR1 as similar protein support. A distance tree was created and bootstrap values were calculated using PAUP* 4.0 from the above alignment (Sinauer Associates, http://www.sinauer.com/).

### Southern blot analysis.

Genomic DNA was isolated as described [13] from the terminal flag leaves of adult plants segregating for *rmr1, rmr6,* and *mop1* mutants and heterozygous siblings as well as *Pl′* and *Pl-Rh* plants as assayed by anther pigmentation [7,8,10,13]. Restriction digest and subsequent Southern blots were carried out as previously described [13], using the restriction enzymes listed in Figure 4 (New England Biolabs, http://www.neb.com/). The probes specific to *pl1* are shown in Figure 4; the 45S and centromere probes are as described [13].

### Small RNA northern blots.

Small RNAs were prepared from 10-mm immature ear tissue and used to generate small RNA northern blots as previously described [63]. In Figure 4D the small RNAs were run with a 27-bp DNA oligonucleotide containing *doppia* sequence that hybridized with the riboprobe used to identify the small RNAs. The riboprobe was synthesized as described [63] from a plasmid containing the region denoted probe B in Figure 4A linearized at an AseI site so as to contain only *doppia* sequence.

### *Pl′* establishment tests.

Establishment of the *Pl′* state in *rmr1* mutants was assayed essentially as described previously [11]. When the T6–9 interchange pair is heterozygous with structurally normal chromosomes, the plants display ∼50% pollen sterility due to meiotic-segregation-induced aneuploidy in the resulting gametes. Pollen sterility was assayed in the field using a pocket microscope. *rmr1* mutants were crossed to *Pl-Rh/Pl-Rh* A619 or A632 inbreds (Table S1), and the resultant progeny were scored with respect to *Pl1-Rhoades* expression state.

## Supporting Information

Dataset S1 Sequence Information for *rmr1* Progenitor and Mutant Alleles (42 KB PDF)

Figure S1 *rmr1* Does Not Affect *Pl1-Rhoades* Transcription Rates In vitro assays with isolated husk nuclei show no differences in *pl1* transcription rates between *rmr1* mutants and non-mutant heterozygotes.
(A) In vitro radiolabeled RNAs corresponding to specific genes from isolated husk nuclei of sibling plants detected with slotblot hybridizations *(pBS*, bacterial plasmid DNA; *pl1*, *purple plant1*; *b1, colored plant1*; *a1, anthocyaninless1*; *uq, ubiquitin2)*.
(B) Quantification of relative mean transcription rates from five independent sets of *+/rmr1–1* (open) and *rmr1–1/rmr1–1* (closed) siblings (± standard error of the mean) showing no significant difference between *pl1* transcription rates.
(C) In vitro radiolabeled RNAs from isolated husk nuclei of *rmr1–3* mutants and heterozygous siblings used to generate quantification in Figure 1A.
(708 KB TIF)

Figure S2 RMR1 Is Structurally Related to Other Snf2 Proteins Multiple species alignment of RMR1 helicase domain with other known and predicted Snf2 proteins.
(101 KB TIF)

Figure S3 *rmr1* Affects DNA Methylation Patterns at *Pl1-Rhoades*
Additional Southern blots comparing the DNA methylation status of the *Pl1-Rhoades* upstream region in *rmr1–1* mutants and heterozygous siblings.
(A) Genomic digests of an *rmr1–1* mutant (−) and heterozygous sibling (+) using methylation-sensitive restriction enzymes in concert with BsrI, hybridized with probe A (Figure 4A). These results were used to generate the methylation profile shown in Figure 4A.
(B) Blot hybridized with probe A comparing *rmr1–1* mutants to heterozygous siblings with respect to methylation at a PspGI site.
(2.4 MB TIF)

Figure S4 *rmr6* Affects DNA Methylation Patterns at *Pl1-Rhoades*
Southern blots comparing the DNA methylation status of the *Pl1-Rhoades* upstream region in *rmr6–1* mutants and heterozygous siblings.
(A) Methylation profile similar to that shown in Figure 4A showing sites at the *Pl1-Rhoades* locus hypomethylated (open circle) in a *rmr6–1* mutant as compared to heterozygous siblings.
(B and C) Blots shown in (B) and (C) were used to generate this profile and are analogous to the blots shown for *rmr1* mutants in Figure S3A and S3B, respectively.
(2.2 MB TIF)

Figure S5 *mop1* Affects DNA Methylation Patterns at *Pl1-Rhoades*
Southern blots comparing the DNA methylation status of the *Pl1-Rhoades* upstream region in *mop1–1* mutants and heterozygous siblings.
(A) Methylation profile similar to that shown in Figure 4A showing sites at the *Pl1-Rhoades* locus hypomethylated (open circle) in a *mop1–1* mutant as compared to heterozygous siblings.
(B and C) Blots shown in (B) and (C) were used to generate this profile and are analogous to the blots shown for *rmr1* mutants in Figure S3A and S3B, respectively.
(2.2 MB TIF)

Figure S6 *rmr1* Affects Abundance of *doppia* Small RNAs Additional small RNA northern blots showing that *doppia* small RNAs of both sense and antisense orientations are absent in *rmr1* mutants. Small RNA northern blots were probed with probe B (Figure 4A) in both the sense (A) and antisense (B) orientations, showing small RNAs (∼26 nt) with *doppia* sequence similarity are present in sense and antisense orientation in *rmr1–1* heterozygotes (+) and are lost in *rmr1–1* mutants. DNA oligonucleotides (22 and 21 nt) used as sizing standards are also shown.
(1.3 MB TIF)

Figure S7 Mutations at *rmr1* Do Not Affect Genome-Wide Methylation Levels Southern blots show that *rmr1* mutations do not affect methylation levels at centromeric sequences and 45S ribosomal DNA repeats. Genomic DNA from four *rmr1–3* mutants and non-mutant siblings digested with BstNI (“B” lanes) and a CNG methylation-sensitive enzyme, PspGI (“P” lanes), which has the same recognition site, probed with (A) radiolabeled centromere sequence and (B) 45S repeat sequence. The comparison between the PspGI digests in mutant and non-mutant individuals reveals no gross methylation differences.
(6.2 MB TIF)

Figure S8 *Pl-Rh* and *Pl′* States Have Identical DNA Methylation Patterns Additional Southern blots show no changes in *Pl1-Rhoades* methylation status between the *Pl-Rh* and *Pl′* states.
(A) The methylation status of upstream PspGI sites is compared for *Pl′*/*Pl′* and *Pl-Rh*/*Pl-Rh* plants with the *Pl1-Rhoades* allele introgressed (>98%) into distinct A619 and A632 backgrounds via hybridization with probe A. The blot reveals no methylation differences at this site between the two *Pl1-Rhoades* regulatory states. The “C” lanes indicate control lanes where the digest was carried out with BstNI, a methylation-insensitive restriction enzyme.
(B) Analogous to blot shown in Figure 4C, though the plants are from a different background (A619 introgression) than the plants used in Figure 4C (A632 introgression), showing that there are no methylation differences at the StuI site in either background.
(1.9 MB TIF)

Figure S9 *rmr1* Is Expressed in Rapidly Dividing Tissues RT-PCR expression profile shows *rmr1* is expressed primarily in tissues with high mitotic index. Tissue samples represented in the analysis include seedling leaf, adult leaf, shoot apical meristem, immature tassel, and immature ear. RT-PCR was carried out using primers that span the first and second introns of *rmr1*.
(358 KB TIF)

Protocol S1 Additional Methods Used to Generate Supporting Pieces of Data (47 KB DOC)

Table S1 *rmr1* Is Not Required to Establish *pl1* Paramutation Test cross results measuring acquisition of paramutagenicity by *Pl-Rh* in *T Pl-Rh rmr1–2*/+ *Pl′ rmr1–1* plants. Genetic assay used for this experiment is detailed in Results and Figure 5. Table details individual anther phenotypes using a 1–7 graded anther color score for specific test cross progeny. Progeny structural genotypes refer to the presence or absence of the reference T6–9 interchange chromosome.
(127 KB DOC)

Table S2 *rmr1* Is Not Required to Establish *b1* Paramutation Table details individual progeny plant phenotypes from crosses of *rmr1*/*rmr1; B-I*/*B′* plants to *Rmr1 b1* testers. Two different mutant *rmr1* alleles are assayed. The B-I and B′ plant phenotypes represent darkly pigmented and light or variegated pigment, respectively. With four exceptions, 206 test cross progeny had a B′ phenotype.
(38 KB DOC)

Table S3 Two New *rmr* Mutations Are Alleles of *rmr1*
Table details individual progeny anther phenotypes graded using a 1–7 anther color score from crosses designed to test genetic complementation of various *rmr* mutations.
(70 KB DOC)

Table S4 Oligonucleotides Used in This Work (45 KB DOC)

## Abbreviations

EMS: ethyl methanesulfonate
RdDM: RNA-directed DNA methylation
RT-PCR: reverse transcriptase PCR
siRNA: small interfering RNA
