# Supplementary material for: A Novel Snf2 Protein Maintains trans-Generational Regulatory States Established by Paramutation in Maize
Source: PLoS Biol. 2007 Oct 16;5(10):e275. doi: 10.1371/journal.pbio.0050275 (PMC2020503; doi:10.1371/journal.pbio.0050275)
Supplement: Supplementary file 1 [file pbio.0050275.sd001.pdf]

>Rmr1-Progenitor allele | genomic | 6610 bp
ATGGATCGCGCCACGCCGCGCGTTTGCGGCCGCCGCGGCGTATCCCAAGCGGCGGTGGAAGCTG
CGCCGTCCTCCTCCCGCGCGCGCCGCCGCGATAAGGCGCCGGCCGTTGTCATGGACCTTGGCGA
CGACGACTGCGGCGGTGGCGGGGCCAGGAAGACGGTTGGTGGCGCTGCAGGTAGGTGCGAGGGA
TCCACGAAGGCTCCTTCGCCTATGCTGCCGCCGATGATGGTGCCGGCGGGAGCGGTGGCGCTGC
GGACACGATCGAGGAGGCGGGCGATGCTGGCGGCGGCAGTGGTGGAAGAGGCGCCAACGAAGAA
GAAGAAGAAGGAAGGAGCGATCCCAGATGCCGCGGAGGCAACCCGTGGCCACGGGAGCAAGGCC
GCTGCGACCTCGATGGCGACGTCGAGCCATAAGCGGCGCGCTGGGACCTCGAGGTCGACGTCGA
GAGATAAGCGGCGCGCGCGCTCGGGACGTGCCTCGGAGCCAGCTCGCGTGGGCCGCGCGCGCAA
GCGCAAAAGGAACGAGTTGGAGGCCCCCGCTCGGAGAGAACGTGTGAAGGCGCCATGTGTCAGT
GAAAGTGATGACAACAGCGGGCGAGGCGATGACGCTTCTCATGACGGGGATGCGGAGCCTCGCG
GTGGGGTCGCCATTGGCACTGATCTGGTTAACGGGGACCATCCGGCAGCTAAAGGTGAGGATCA
TATTAAGAGAAGAGTGCTGTGAATTTGAATGATGACTTGGTCTCATGTAATGCAGAGGTAGTGG
AAGGTGCTGGTGACGAGGACACAGGGGACGGAGGGAACAGTGGCCTGGCATCGACTGCTGATGT
GGTTGCTGAGGAGATGGCACCCTTTGAAGATGACTACGATGATGAGATGTTGGAGGAGCAGCTT
GTTGGAGATGTGATTCGTGCTTACAGTAATGGCAGAAACTTAGATTCAGATGGAGTGGATTGGG
AGGCAGAGGATGAGATGGAGTTCAATGACGATGCTGACAATAGTGATTTTATGGATGATGCTGA
CGATAGTGATTTTATGGATGATGCTTATGAAGGTGGCAATTCCAAACCAATTCAAAATCATGCT
AAGTTGGAAATACAAGACTGGGTGAACCAGAAAGTTGTTTTGAGTGGAGGGAGGTGTGAGGTGA
GGGGCGAGGGGGATCTGGAGGAAGAATTGGGTGTGGGAAAGGAAGCAGATGAGGAGGACGTGGA
ACCAAAGAGTGAAGCAGCTCCAGGTTCTGATAAAAGGGTCTTGCAGTTAGAAATCCTAGGTTCC
GATGAGGAAATCAAGGTGCTTGAAAATATGAGTAGTGCCCCATCCAGGAAGGCGTCAGTTCAAT
CGAAGTTACCAACTATTCCATCTTGTGTTGCATGGAGAACCCGATCATCATGGGGGGTAAATCA
AGATAGACTATCGTACGATACATATTTTGAGGAATTATCTGATGAGCCAAAAGAGGATGATGAT
GATACAGAGGTGGAACTTGATGAAGTTGAGGATGACAACAATGACGATGACAGTAGTGATGCTT
ATGATAAAGATGACGAAGAGAAGGAGGAAGAGGAAGAAGAGGCTGAAAGAAGAAAACTTAATAA
CAGGATTTGCACATCCGATGAAGACATGATCAATATTACTGTTCCTACATCAAGATATGATATG
TTTAAGAAAAAAAATTCCTCAAGATATGATATTGAGTGGGTGGAGGATGAAGATGCAAGTGTTG
ATATGTTACAGCCAGTTTCCTTTAAGAAAGATAGCAGCTGGAAGCCTGTGGCTGTTGGCAACGA
CACATTTACTGAGCAACAAAAGCGATCACGATTTACTTGGGAGCTTGAGAGGAGGAAAAAGCTT
AAGCTTGAGATGAAGACAAATCCTTTGCATGAGCGGGATTTGGACTCAGATCCGAACTCATCAG
GTTCTGACCAGATCAGAAAGTATGGTTTCAAAAGTGATGGGAGTCATAAAGTTGATAGGAAAAA
GAAGCATACATCGCCCAAATCGGGCAAGAAACCCAGCAGCGCAATCATACTAAAGCGGCAGTCT
CTTTTGAAGCTTTTGGTAGATAAAATGAGTGGTGATAAAAGTTTAGCATCTTTTCCATTTGATC
AGAATCCTCAGCTTCAGTTTATTTTCAAAGAAATGCATCCATTGGTATTTTCATTTGGAGATGA
AGATCTAGTAGCAGCTGACAGGCCAGAGCAAGATGTTGGATTGGATATGTTATGGGCTGACTTT
GACTTTGCTTTAGAGTCTGAGAATATCGGTACTTATTATGATGATGAGGTACATTCAGTACTGG
TTTTCATTTTATTTTTATCTATCAAAACACATGCAGGTATAAATATGTGTGGTTTATTTATTTG
AACACTGACTCTACAACTAGAACTATGGTAAAATTAACAATGTCCAACTGGAGATGCACTACCA
TGTTATTCTCAGCAAAAAGTTTGTCAATGGCGACAAACGCAAAAAGTTTGATGAAACAATCTCG
ATCAAACCAAAAAAACTCTCATTATTCACCATCTGGCACAAGGGCAAAGATGCAGGAGATGCCC
CTCTAGGGAGCAGAACTCCAGCTATTTTTGGAGATGCACCATCAAACACATATCAGCTTTGTCA
TTTCCAAATTGCCCATGCTCCAAGGGTGATGATCGAATTAAGCCCATTTTGTTCCTCACCATTT
TTTTCAACGGACCTTGTGAAACCTAACCATCAACCCTTGCTTCTCCCCTTGCCGATCATTCATC
AAAGGAAACATCATCTATCTGTGGAGGACCTGCAGGCTGCAGCAGTCTAAACCAGAATTCTCAT
GCAAAAACACACAATGTGAGCAGGTGATTGATCATCTCAGCTTGATCACAAAGAGCACAGCAGG

TAGGGTGCAGAAGACTCATCATGCATGCTGATCGGCAGTCCATTACCTATTTTGAGTAAGTAAC
CACAAAAGAACCAACACTTCCTGGGCCCCAAGATTTTCAAATTCTTTCCCAAGACCCAAGAGGA
CAGAACCATGAAATAAACCCTTATCAACAGACTTGCCTCAATCATCTACATCTATCGTTAACAT
AACTAACAAGAGAAGAAATAAACCATTTGAGCTCACTTTGATAGTGCAACACTTCTGTGAACAA
TATGTGCATGTGAAATGTAGCCTCCTTTTCTCAGAATGCATATATCTGTGGTGTTTGTCAAGGG
CCTCACACTGCTGTCGTGTCCATAAAGAAAGCACAGGAATCAACAATCCTTCTTCGGGAACACT
ATTGAAGCTAGAACCCAGAAATGTACAGAAAAAACTATTAGGGCGTGGAAATTCTTTCTGCATT
TTCTTCAGTTTGATCTGGCTCTATTGTTTCTCAGATCATGTCTACGTCATGGTAGTTTTTTTT
ATCGAATGCGCAGGAGAGATGCGCATCATTATATTAAGAGATGAAAAGGTCCAAAATAGACCAG
CACAAGATAGTAGAAAAGGCCCTTTATGGTGGCCAAAAAGTAAGATACAGAAAATGATCCATTA
AATCAATCATACTATAAATCCGACCGAAGAGGGTCGGCAAGGTAGTTTGCAGCTAATGTGCAGG
AAACGTCGTTTCCCAGAATAGAGTAACTTTGTAAACATTAGAAATGTTTTTTTTCAGGTCAAGA
AATCTAGTTCACTTAAAGGAGTTACTATAGAATAGTTTATAACCATTATATTAAGAAGAGATGA
AAAGGTCCAAAATAGACCAGCACAAGATAGTACAAAAGGCCCTTTATGGCGGAGTAAGATACAG
AAAATGATCCATTAAATCAATCCTACTATAAATCCGACCGAAGAGGGGCAGCAAGGTAGTTTGC
AGCTAATGTGCAGGAAACGTCGTTTCCCAGAATAGAGTAACTTTGTAAACATTAGAAATGTTTT
TTTAGGTCAAGAACTCTAGTTCACTTAAAGGAGTTACTATAGAATAGTTTATAACCATTAAGGG
GCAATTCAGATAGCCTCAGAAGGTCAAGAAAACTTTATCATAGCCTGTGTGAATGTTTAGACAA
AAAGGAAGCAGAAATGTTTGTTCTTTGTCAAAAGTTTCATCATCAGTGTGTTTCATTTAATGAT
TTCCTTTTGTCCCAACATTAATGACTCAGTTACATGTTGTTTTTGCGAATGGAATTTTCTAACT
TGGCAATGTTACTCAAACATATGTAGTATTCTGCATATCTGATACCACAGGTTGATTCCTACTC
TTAATTCGGCAATCAACATAAATAGTTTCATCTTTTAGAAACTAGACACCCCCTACTGCTTTCA
TGTAAAAGTTATATAGCATTTCAAGGTCGTGCGTCCATGATATACTACTCGATTTTTAATGTTT
ATTTTCTTGAATGCAAGAGTTTGTACCATTGCAGATACTTTGATTTGCCACTGTGAGAATGACT
AAATGAACTTATTAGCTTATGTTGTATTGTAGTGTCAAGAAGGCAATCAACTAGATTTTTCTCT
TGCCTCAGTAACACCCTGTTCTCGTGGGAAGCATGAATTTGTTATTGATGATCAAATAGGGATC
AGATGCAAATACTGTTCGTTGGTAAACCTGGAGATCAAATTCATGTTTCCATCACTGGTAAGCT
TTATTATGGTCATAAATCATGACATCTATTCTACATAAATTTGGTCATTTAAGTCATCATTTTC
TTTTAGGTGTCAGTGTTTGGCGAGAAATCAGCATGGCCAAATGACAAAGGCGTGAAGAATACAC
TGATGTTTCATGATCTTTATGAACAAGGAGTCAATGACACTGAACAATCTCAAGATATTCATCA
ATATGGAACGGTGTGGAATCTTATTCCAGGGGTCATCAGTACTATGTATGAGCATCAGCGTGAA
GCATTTGAATTTATGTGGACAAATTTAGTTGGTGATATTAGACTTGATGAGATAAAGCATGGAG
CAAAACCTGATGTTGTTGGTGGATGTGTTATCTGTCATGCTCCTGGAACAGGAAAGACACGATT
AGCTATTGTATTTATCCAGACATACATGAAAGTGTTTCCAGACTGTCGGCCAGTGATTATTGCA
CCACGTGGTATGCTCTTTGCTTGGGATGAGGAATTTAAGAAATGGAATGTTGATGTTCCTTTTC
ATATACTAAACACAACTGATTACACTGGAAAAGAGGACCGGGACATATGCAAGTTAATAAAGAA
AGAACATAGGACAGAAAAGTTGACAAGACTAGTCAAACTGCTTTCATGGAACAAAGGCCATGGT
ATTCTTGGAATAAGTTATGGTCTGTACACAAAACTGACCTCTGAAAAACCTGGCTGCACAGAAG
AAAACAAAGTAAGAAGCATTCTTCTTGATAACCCTGGCTTACTTGTTCTTGATGAAGGACATAC
ACCTAGGAATGAGCGCAGTGTTATGTGGAAAACTCTAGGAAATGTGAAAACTGAGAAGCGTATA
ATTTTATCTGGAACTCCTTTTCAGAACAATTTTCTTGAGCTTTACAACATTCTTTGTCTGGTAA
GGCCTAGATTTGGTGAAATGTTTTTGACGAAGTCAAGAGTAGGTCGAAGACATTATGTCTCAAA
AAAGCAAAAGGATAAGTTTTCTGATAAATATGAAAAGGGTGTTTGGGCATCACTGACTAGCAAT
GTAACTGATGATAATGCGGAGAAAGTAAGATCAATATTGAAACCATTTGTTCATATACATAATG
GCAATATTCTTCGAACTCTTCCAGGACTCAGGGAGAGTGTAATTATTTTGAAGCCTCTTCCCCT
TCAAAAGAGTATCATTAAAAAGGTGGAAAACATTGGTTCTGGTAACAACTTCGAACATGAATAT

GTCATTTCTTTAGCTTCTACACACCCTTCCCTTGTAACCGCCATTAACATGTCTGAGGAGGAAG
CTTCACTTATTGATAAACCTATGCTTGCTAAAGTGAGATCAAATCCATATGAAGGGGTAAAAAC
AAGATTTGTGATCGAAGTTGTTCGTTTGTCTGAAGCATTAAGAGAGAAGGTTTTGATTTTTAGC
CAATTTATTCAGCCTCTAGAGTTGATTAAAGAGCATCTTCGCAAGTTCTTCAAATGGAGAGAAG
GGAAAGAAATTCTTCAAATGGATGGAAAGATCCTTCCAAGATATCGCCAGGCTTCCATTGAAGC
CTTCAATAATCCAAATAATGATTCCAGGGTGTTACTTGCATCTACAAGAGCATGCTGTGAAGGG
ATTAGCTTGACAGGTGCTTCAAGAATTGTGCTTCTAGATGTTGTTTGGAACCCAGCTGTTGGAA
GGCAAGCCATCAGCAGAGCATTTAGGATAGGTCAGAAGAAATTTGTATATACATATAATTTGAT
AACTTATGGAACAGGTGAAGGTGACAAATATGATAGGCAAGCAGAAAAGGATCACTTATCCAAG
TTGGTCTTCTCTACAGAAGACGAGTTCAATAATGTTAGGAACATGTTATCTAAAGCTGAAATGG
AGCACTGTTCTAAGTTTATCTCAGAAGATAAAGTTTTGGAGGAGATGACTTCCCACGATCAACT
TAAAGGAATGTTTTTGAAGATCCATTATCCACCAACTGAGTCAAACATTGTCTATAGTTACAAT
CAAATTGCTACTGAGTGA

>Rmr1-Progenitor allele | coding sequence | 4308 bp
ATGGATCGCGCCACGCCGCGCGTTTGCGGCCGCCGCGGCGTATCCCAAGCGGCGGTGGAAGCTG
CGCCGTCCTCCTCCCGCGCGCGCCGCCGCGATAAGGCGCCGGCCGTTGTCATGGACCTTGGCGA
CGACGACTGCGGCGGTGGCGGGGCCAGGAAGACGGTTGGTGGCGCTGCAGGTAGGTGCGAGGGA
TCCACGAAGGCTCCTTCGCCTATGCTGCCGCCGATGATGGTGCCGGCGGGAGCGGTGGCGCTGC
GGACACGATCGAGGAGGCGGGCGATGCTGGCGGCGGCAGTGGTGGAAGAGGCGCCAACGAAGAA
GAAGAAGAAGGAAGGAGCGATCCCAGATGCCGCGGAGGCAACCCGTGGCCACGGGAGCAAGGCC
GCTGCGACCTCGATGGCGACGTCGAGCCATAAGCGGCGCGCTGGGACCTCGAGGTCGACGTCGA
GAGATAAGCGGCGCGCGCGCTCGGGACGTGCCTCGGAGCCAGCTCGCGTGGGCCGCGCGCGCAA
GCGCAAAAGGAACGAGTTGGAGGCCCCCGCTCGGAGAGAACGTGTGAAGGCGCCATGTGTCAGT
GAAAGTGATGACAACAGCGGGCGAGGCGATGACGCTTCTCATGACGGGGATGCGGAGCCTCGCG
GTGGGGTCGCCATTGGCACTGATCTGGTTAACGGGGACCATCCGGCAGCTAAAGGGGTAGTGGA
AGGTGCTGGTGACGAGGACACAGGGGACGGAGGGAACAGTGGCCTGGCATCGACTGCTGATGTG
GTTGCTGAGGAGATGGCACCCTTTGAAGATGACTACGATGATGAGATGTTGGAGGAGCAGCTTG
TTGGAGATGTGATTCGTGCTTACAGTAATGGCAGAAACTTAGATTCAGATGGAGTGGATTGGGA
GGCAGAGGATGAGATGGAGTTCAATGACGATGCTGACAATAGTGATTTTATGGATGATGCTGAC
GATAGTGATTTTATGGATGATGCTTATGAAGGTGGCAATTCCAAACCAATTCAAAATCATGCTA
AGTTGGAAATACAAGACTGGGTGAACCAGAAAGTTGTTTTGAGTGGAGGGAGGTGTGAGGTGAG
GGGCGAGGGGGATCTGGAGGAAGAATTGGGTGTGGGAAAGGAAGCAGATGAGGAGGACGTGGAA
CCAAAGAGTGAAGCAGCTCCAGGTTCTGATAAAAGGGTCTTGCAGTTAGAAATCCTAGGTTCCG
ATGAGGAAATCAAGGTGCTTGAAAATATGAGTAGTGCCCCATCCAGGAAGGCGTCAGTTCAATC
GAAGTTACCAACTATTCCATCTTGTGTTGCATGGAGAACCCGATCATCATGGGGGGTAAATCAA
GATAGACTATCGTACGATACATATTTTGAGGAATTATCTGATGAGCCAAAAGAGGATGATGATG
ATACAGAGGTGGAACTTGATGAAGTTGAGGATGACAACAATGACGATGACAGTAGTGATGCTTA
TGATAAAGATGACGAAGAGAAGGAGGAAGAGGAAGAAGAGGCTGAAAGAAGAAAACTTAATAAC
AGGATTTGCACATCCGATGAAGACATGATCAATATTACTGTTCCTACATCAAGATATGATATGT
TTAAGAAAAAAAATTCCTCAAGATATGATATTGAGTGGGTGGAGGATGAAGATGCAAGTGTTGA
TATGTTACAGCCAGTTTCCTTTAAGAAAGATAGCAGCTGGAAGCCTGTGGCTGTTGGCAACGAC
ACATTTACTGAGCAACAAAAGCGATCACGATTTACTTGGGAGCTTGAGAGGAGGAAAAAGCTTA
AGCTTGAGATGAAGACAAATCCTTTGCATGAGCGGGATTTGGACTCAGATCCGAACTCATCAGG
TTCTGACCAGATCAGAAAGTATGGTTTCAAAAGTGATGGGAGTCATAAAGTTGATAGGAAAAAG
AAGCATACATCGCCCAAATCGGGCAAGAAACCCAGCAGCGCAATCATACTAAAGCGGCAGTCTC

TTTTGAAGCTTTTGGTAGATAAAATGAGTGGTGATAAAAGTTTAGCATCTTTTCCATTTGATCA
GAATCCTCAGCTTCAGTTTATTTTCAAAGAAATGCATCCATTGGTATTTTCATTTGGAGATGAA
GATCTAGTAGCAGCTGACAGGCCAGAGCAAGATGTTGGATTGGATATGTTATGGGCTGACTTTG
ACTTTGCTTTAGAGTCTGAGAATATCGGTACTTATTATGATGATGAGTGTCAAGAAGGCAATCA
ACTAGATTTTTCTCTTGCCTCAGTAACACCCTGTTCTCGTGGGAAGCATGAATTTGTTATTGAT
GATCAAATAGGGATCAGATGCAAATACTGTTCGTTGGTAAACCTGGAGATCAAATTCATGTTTC
CATCACTGGTGTCAGTGTTTGGCGAGAAATCAGCATGGCCAAATGACAAAGGCGTGAAGAATAC
ACTGATGTTTCATGATCTTTATGAACAAGGAGTCAATGACACTGAACAATCTCAAGATATTCAT
CAATATGGAACGGTGTGGAATCTTATTCCAGGGGTCATCAGTACTATGTATGAGCATCAGCGTG
AAGCATTTGAATTTATGTGGACAAATTTAGTTGGTGATATTAGACTTGATGAGATAAAGCATGG
AGCAAAACCTGATGTTGTTGGTGGATGTGTTATCTGTCATGCTCCTGGAACAGGAAAGACACGA
TTAGCTATTGTATTTATCCAGACATACATGAAAGTGTTTCCAGACTGTCGGCCAGTGATTATTG
CACCACGTGGTATGCTCTTTGCTTGGGATGAGGAATTTAAGAAATGGAATGTTGATGTTCCTTT
TCATATACTAAACACAACTGATTACACTGGAAAAGAGGACCGGGACATATGCAAGTTAATAAAG
AAAGAACATAGGACAGAAAAGTTGACAAGACTAGTCAAACTGCTTTCATGGAACAAAGGCCATG
GTATTCTTGGAATAAGTTATGGTCTGTACACAAAACTGACCTCTGAAAAACCTGGCTGCACAGA
AGAAAACAAAGTAAGAAGCATTCTTCTTGATAACCCTGGCTTACTTGTTCTTGATGAAGGACAT
ACACCTAGGAATGAGCGCAGTGTTATGTGGAAAACTCTAGGAAATGTGAAAACTGAGAAGCGTA
TAATTTTATCTGGAACTCCTTTTCAGAACAATTTTCTTGAGCTTTACAACATTCTTTGTCTGGT
AAGGCCTAGATTTGGTGAAATGTTTTTGACGAAGTCAAGAGTAGGTCGAAGACATTATGTCTCA
AAAAAGCAAAAGGATAAGTTTTCTGATAAATATGAAAAGGGTGTTTGGGCATCACTGACTAGCA
ATGTAACTGATGATAATGCGGAGAAAGTAAGATCAATATTGAAACCATTTGTTCATATACATAA
TGGCAATATTCTTCGAACTCTTCCAGGACTCAGGGAGAGTGTAATTATTTTGAAGCCTCTTCCC
CTTCAAAAGAGTATCATTAAAAAGGTGGAAAACATTGGTTCTGGTAACAACTTCGAACATGAAT
ATGTCATTTCTTTAGCTTCTACACACCCTTCCCTTGTAACCGCCATTAACATGTCTGAGGAGGA
AGCTTCACTTATTGATAAACCTATGCTTGCTAAAGTGAGATCAAATCCATATGAAGGGGTAAAA
ACAAGATTTGTGATCGAAGTTGTTCGTTTGTCTGAAGCATTAAGAGAGAAGGTTTTGATTTTTA
GCCAATTTATTCAGCCTCTAGAGTTGATTAAAGAGCATCTTCGCAAGTTCTTCAAATGGAGAGA
AGGGAAAGAAATTCTTCAAATGGATGGAAAGATCCTTCCAAGATATCGCCAGGCTTCCATTGAA
GCCTTCAATAATCCAAATAATGATTCCAGGGTGTTACTTGCATCTACAAGAGCATGCTGTGAAG
GGATTAGCTTGACAGGTGCTTCAAGAATTGTGCTTCTAGATGTTGTTTGGAACCCAGCTGTTGG
AAGGCAAGCCATCAGCAGAGCATTTAGGATAGGTCAGAAGAAATTTGTATATACATATAATTTG
ATAACTTATGGAACAGGTGAAGGTGACAAATATGATAGGCAAGCAGAAAAGGATCACTTATCCA
AGTTGGTCTTCTCTACAGAAGACGAGTTCAATAATGTTAGGAACATGTTATCTAAAGCTGAAAT
GGAGCACTGTTCTAAGTTTATCTCAGAAGATAAAGTTTTGGAGGAGATGACTTCCCACGATCAA
CTTAAAGGAATGTTTTTGAAGATCCATTATCCACCAACTGAGTCAAACATTGTCTATAGTTACA
ATCAAATTGCTACTGAGTGA

>Rmr1-A619 | genomic | 6610 bp
ATGGATCGCGCCACGCCGCGCGTTTGCGGCCGCCGCGGCGTATCCCAAGCGGCGGTGGAAGCTG
CGCCGTCCTCCTCCCGCGCGCGCCGCCGCGATAAGGCGCCGGCCGTTGTCATGGACCTTGGCGA
CGACGACTGCGGCGGTGGCGGGGCCAGGAAGACGGTTGGTGGCGCTGCAGGTAGGTGCGAGGGA
TCCACGAAGGCTCCTTCGCCTATGCTGCCGCCGATGATGGTGCCGGCGGGAGCGGTGGCGCTGC
GGACACGATCGAGGAGGCGGGCGATGCTGGCGGCGGCAGTGGTGGAAGAGGCGCCAACGAAGAA
GAAGAAGAAGGAAGGAGCGATCCCAGATGCCGCGGAGGCAACCCGTGGCCACGGGAGCAAGGCC
GCTGCGACCTCGATGGCGACGTCGAGCCATAAGCGGCGCGCTGGGACCTCGAGGTCGACGTCGA

GAGATAAGCGGCGCGCGCGCTCGGGACGTGCCTCGGAGCCAGCTCGCGTGGGCCGCGCGCGCAA
GCGCAAAAGGAACGAGTTGGAGGCCCCCGCTCGGAGAGAACGTGTGAAGGCGCCATGTGTCAGT
GAAAGTGATGACAACAGCGGGCGAGGCGATGACGCTTCTCATGACGGGGATGCGGAGCCTCGCG
GCGGGGTCGCCATTGGCACTGATCTGGTTAACGGGGACCATCCGGCAGCTAAAGGTGAGGATCA
TATTAAGAGAAGAGTGCTGTGAATTTGAATGATGACTTGGTCTCATGTAATGCAGAGGTAGTGG
AAGGTGCTGGTGACGAGGACACAGGGGACGGAGGGAACAGTGGCCTGGCATCGACTGCTGATGT
GGTTGCTGAGGAGATGGCACCCTTTGAAGATGACTACGATGATGAGATGTTGGAGGAGCAGCTT
GTTGGAGATGTGATTCGTGCTTACAGTAATGGCAGAAACTTAGATTCAGATGGAGTGGATTGGG
AGGCAGAGGATGAGATGGAGTTCAATGACGATGCTGACAATAGTGATTTTATGGATGATGCTGA
CGATAGTGATTTTATGGATGATGCTTATGAAGGTGGCAATTCCAAACCAATTCAAAATCATGCT
AAGTTGGAAATACAAGACTGGGTGAACCAGAAAGTTGTTTTGAGTGGAGGGAGGTGTGAGGTGA
GGGGCGAGGGGGATCTGGAGGAAGAATTGGGTGTGGGAAAGGAAGCAGATGAGGAGGACGTGGA
ACCAAAGAGTGAAGCAGCTCCAGGTTCTGATAAAAGGGTCTTGCAGTTAGAAATCCTAGGTTCC
GATGAGGAAATCAAGGTGCTTGAAAATATGAGTAGTGCCCCATCCAGGAAGGCGTCAGTTCAAT
CGAAGTTACCAACTATTCCATCTTGTGTTGCATGGAGAACCCGATCATCATGGGGGGTAAATCA
AGATAGACTATCGTACGATACATATTTTGAGGAATTATCTGATGAGCCAAAAGAGGATGATGAT
GATACAGAGGTGGAACTTGATGAAGTTGAGGATGACAACAATGACGATGACAGTAGTGATGCTT
ATGATAAAGATGACGAAGAGAAGGAGGAAGAGGAAGAAGAGGCTGAAAGAAGAAAACTTAATAA
CAGGATTTGCACATCCGATGAAGACATGATCAATATTACTGTTCCTACATCAAGATATGATATG
TTTAAGAAAAAAAATTCCTCAAGATATGATATTGAGTGGGTGGAGGATGAAGATGCAAGTGTTG
ATATGTTACAGCCAGTTTCCTTTAAGAAAGATAGCAGCTGGAAGCCTGTGGCTGTTGGCAACGA
CACATTTACTGAGCAACAAAAGCGATCACGATTTACTTGGGAGCTTGAGAGGAGGAAAAAGCTT
AAGCTTGAGATGAAGACAAATCCTTTGCATGAGCGGGATTTGGACTCAGATCCGAACTCATCAG
GTTCTGACCAGATCAGAAAGTATGGTTTCAAAAGTGATGGGAGTCATAAAGTTGATAGGAAAAA
GAAGCATACATCGCCCAAATCGGGCAAGAAACCCAGCAGCGCAATCATACTAAAGCGGCAGTCT
CTTTTGAAGCTTTTGGTAGATAAAATGAGTGGTGATAAAAGTTTAGCATCTTTTCCATTTGATC
AGAATCCTCAGCTTCAGTTTATTTTCAAAGAAATGCATCCATTGGTATTTTCATTTGGAGATGA
AGATCTAGTAGCAGCTGACAGGCCAGAGCAAGATGTTGGATTGGATATGTTATGGGCTGACTTT
GACTTTGCTTTAGAGTCTGAGAATATCGGTACTTATTATGATGATGAGGTACATTCAGTACTGG
TTTTCATTTTATTTTTATCTATCAAAACACATGCAGGTATAAATATGTGTGGTTTATTTATTTG
AACACTGACTCTACAACTAGAACTATGGTAAAATTAACAATGTCCAACTGGAGATGCACTACCA
TGTTATTCTCAGCAAAAAGTTTGTCAATGGCGACAAACGCAAAAAGTTTGATGAAACAATCTCG
ATCAAACCAAAAAAACTCTCATTATTCACCATCTGGCACAAGGGCAAAGATGCAGGAGATGCCC
CTCTAGGGAGCAGAACTCCAGCTATTTTTGGAGATGCACCATCAAACACATATCAGCTTTGTCA
TTTCCAAATTGCCCATGCTCCAAGGGTGATGATCGAATTAAGCCCATTTTGTTCCTCACCATTT
TTTTCAACGGACCTTGTGAAACCTAACCATCAACCCTTGCTTCTCCCCTTGCCGATCATTCATC
AAAGGAAACATCATCTATCTGTGGAGGACCTGCAGGCTGCAGCAGTCTAAACCAGAATTCTCAT
GCAAAAACACACAATGTGAGCAGGTGATTGATCATCTCAGCTTGATCACAAAGAGCACAGCAGG
TAGGGTGCAGAAGACTCATCATGCATGCTGATCGGCAGTCCATTACCTATTTTGAGTAAGTAAC
CACAAAAGAACCAACACTTCCTGGGCCCCAAGATTTTCAAATTCTTTCCCAAGACCCAAGAGGA
CAGAACCATGAAATAAACCCTTATCAACAGACTTGCCTCAATCATCTACATCTATCGTTAACAT
AACTAACAAGAGAAGAAATAAACCATTTGAGCTCACTTTGATAGTGCAACACTTCTGTGAACAA
TATGTGCATGTGAAATGTAGCCTCCTTTTCTCAGAATGCATATATCTGTGGTGTTTGTCAAGGG
CCTCACACTGCTGTCGTGTCCATAAAGAAAGCACAGGAATCAACAATCCTTCTTCGGGAACACT
ATTGAAGCTAGAACCCAGAAATGTACAGAAAAAACTATTAGGGCGTGGAAATTCTTTCTGCATT
TTCTTCAGTTTGATCTGGCTCTATTGTTTCTCAGATCATGTCTACGTCATGGTAGTTTTTTTTT

ATCGAATGCGCAGGAGAGATGCACATCATTATATTAAGAGATGAAAAGGTCCAAAATAGACCAG
CACAAGATAGTAGAAAAGGCCCTTTATGGTGGCCAAAAAGTAAGATACAGAAAATGATCCATTA
AATCAATCATACTATAAATCCGACCGAAGAGGGTCGGCAAGGTAGTTTGCAGCTAATGTGCAGG
AAACGTCGTTTCCCAGAATAGAGTAACTTTGTAAACATTAGAAATGTTTTTTTTCAGGTCAAGA
AATCTAGTTCACTTAAAGGAGTTACTATAGAATAGTTTATAACCATTATATTAAGAAGAGATGA
AAAGGTCCAAAATAGACCAGCACAAGATAGTACAAAAGGCCCTTTATGGCGGAGTAAGATACAG
AAAATGATCCATTAAATCAATCCTACTATAAATCCGACCGAAGAGGGGCAGCAAGGTAGTTTGC
AGCTAATGTGCAGGAAACGTCGTTTCCCAGAATAGAGTAACTTTGTAAACATTAGAAATGTTTT
TTTAGGTCAAGAACTCTAGTTCACTTAAAGGAGTTACTATAGAATAGTTTATAACCATTAAGGG
GCAATTCAGATAGCCTCAGAAGGTCAAGAAAACTTTATCATAGCCTGTGTGAATGTTTAGACAA
AAAGGAAGCAGAAATGTTTGTTCTTTGTCAAAAGTTTCATCATCAGTGTGTTTCATTTAATGAT
TTCCTTTTGTCCCAACATTAATGACTCAGTTACATGTTGTTTTTGCGAATGGAATTTTCTAACT
TGGCAATGTTACTCAAACATATGTAGTATTCTGCATATCTGATACCCACAGGTTGATTCCTACTC
TTAATTCGGCAATCAACATAAATAGTTTCATCTTTTAGAAACTAGACACCCCCTACTGCTTTCA
TGTAAAAGTTATATAGCATTTCAAGGTCGTGCGTCCATGATATACTACTCGATTTTTAATGTTT
ATTTTCTTGAATGCAAGAGTTTGTACCATTGCAGATACTTTGATTTGCCACTGTGAGAATGACT
AAATGAACTTATTAGCTTATGTTGTATTGTAGTGTCAAGAAGGCAATCAACTAGATTTTTCTCT
TGCCTCAGTAACACCCTGTTCTCGTGGGAAGCATGAATTTGTTATTGATGATCAAATAGGGATC
AGATGCAAATACTGTTCGTTGGTAAACCTGGAGATCAAATTCATGTTTCCATCACTGGTAAGCT
TTATTATGGTCATAAATCATGACATCTATTCTACATAAATTTGGTCATTTAAGTCATCATTTTC
TTTTAGGTGTCAGTGTTTGGCGAGAAATCAGCATGGCCAAATGACAAAGGCGTGAAGAATACAC
TGATGTTTCATGATCTTTATGAACAAGGAGTCAATGACACTGAACAATCTCAAGATATTCATCA
ATATGGAACGGTGTGGAATCTTATTCCAGGGGTCATCAGTACTATGTATGAGCATCAGCGTGAA
GCATTTGAATTTATGTGGACAAATTTAGTTGGTGATATTAGACTTGATGAGATAAAGCATGGAG
CAAAACCTGATGTTGTTGGTGGATGTGTTATCTGTCATGCTCCTGGAACAGGAAAGACACGATT
AGCTATTGTATTTATCCAGACATACATGAAAGTGTTTCCAGACTGTCGGCCAGTGATTATTGCA
CCACGTGGTATGCTCTTTGCTTGGGATGAGGAATTTAAGAAATGGAATGTTGATGTTCCTTTTC
ATATACTAAACACAACTGATTACACTGGAAAAGAGGACCGGGACATATGCAAGTTAATAAAGAA
AGAACATAGGACAGAAAAGTTGACAAGACTAGTCAAACTGCTTTCATGGAACAAAGGCCATGGT
ATTCTTGGAATAAGTTATGGTCTGTACACAAAACTGACCTCTGAAAAACCTGGCTGCACAGAAG
AAAACAAAGTAAGAAGCATTCTTCTTGATAACCCTGGCTTACTTGTTCTTGATGAAGGACATAC
ACCTAGGAATGAGCGCAGTGTTATGTGGAAAACTCTAGGAAATGTGAAAACTGAGAAGCGTATA
ATTTTATCTGGAACTCCTTTTCAGAACAATTTTCTTGAGCTTTACAACATTCTTTGTCTGGTAA
GGCCTAGATTTGGTGAAATGTTTTTGACGAAGTCAAGAGTAGGTCGAAGACATTATGTCTCAAA
AAAGCAAAAGGATAAGTTTTCTGATAAATATGAAAAGGGTGTTTGGGCATCACTGACTAGCAAT
GTAACTGATGATAATGCGGAGAAAGTAAGATCAATATTGAAACCATTTGTTCATATACATAATG
GCAATATTCTTCGAACTCTTCCAGGACTCAGGGAGAGTGTAATTATTTTGAAGCCTCTTCCCCT
TCAAAAGAGTATCATTAAAAAGGTGGAAAACATTGGTTCTGGTAACAACTTCGAACATGAATAT
GTCATTTCTTTAGCTTCTACACACCCTTCCCTTGTAACCGCCATTAACATGTCTGAGGAGGAAG
CTTCACTTATTGATAAACCTATGCTTGCTAAAGTGAGATCAAATCCATATGAAGGGGTAAAAAC
AAGATTTGTGATCGAAGTTGTTCGTTTGTCTGAAGCATTAAGAGAGAAGGTTTTGATTTTTAGC
CAATTTATTCAGCCTCTAGAGTTGATTAAAGAGCATCTTCGCAAGTTCTTCAAATGGAGAGAAG
GGAAAGAAATTCTTCAAATGGATGGAAAGATCCTTCCAAGATATCGCCAGGCTTCCATTGAAGC
CTTCAATAATCCAAATAATGATTCCAGGGTGTTACTTGCATCTACAAGAGCATGCTGTGAAGGG
ATTAGCTTGACAGGTGCTTCAAGAATTGTGCTTCTAGATGTTGTTTGGAACCCAGCTGTTGGAA
GGCAAGCCATCAGCAGAGCATTTAGGATAGGTCAGAAGAAATTTGTATATACATATAATTTGAT

AACTTATGGAACAGGTGAAGGTGACAAATATGATAGGCAAGCAGAAAAGGATCACTTATCCAAG
TTGGTCTTCTCTACAGAAGACGAGTTCAATAATGTTAGGAACATGTTATCTAAAGCTGAAATGG
AGCACTGTTCTAAGTTTATCTCAGAAGATAAAGTTTTGGAGGAGATGACTTCCCACGATCAACT
TAAAGGAATGTTTTTGAAGATCCATTATCCACCAACTGAGTCAAACATTGTCTATAGTTACAAT
CAAATTGCTACTGAGTGA

>Rmr1-A619 | coding sequence | 4308 bp
ATGGATCGCGCCACGCCGCGCGTTTGCGGCCGCCGCGGCGTATCCCAAGCGGCGGTGGAAGCTG
CGCCGTCCTCCTCCCGCGCGCGCCGCCGCGATAAGGCGCCGGCCGTTGTCATGGACCTTGGCGA
CGACGACTGCGGCGGTGGCGGGGCCAGGAAGACGGTTGGTGGCGCTGCAGGTAGGTGCGAGGGA
TCCACGAAGGCTCCTTCGCCTATGCTGCCGCCGATGATGGTGCCGGCGGGAGCGGTGGCGCTGC
GGACACGATCGAGGAGGCGGGCGATGCTGGCGGCGGCAGTGGTGGAAGAGGCGCCAACGAAGAA
GAAGAAGAAGGAAGGAGCGATCCCAGATGCCGCGGAGGCAACCCGTGGCCACGGGAGCAAGGCC
GCTGCGACCTCGATGGCGACGTCGAGCCATAAGCGGCGCGCTGGGACCTCGAGGTCGACGTCGA
GAGATAAGCGGCGCGCGCGCTCGGGACGTGCCTCGGAGCCAGCTCGCGTGGGCCGCGCGCGCAA
GCGCAAAAGGAACGAGTTGGAGGCCCCCGCTCGGAGAGAACGTGTGAAGGCGCCATGTGTCAGT
GAAAGTGATGACAACAGCGGGCGAGGCGATGACGCTTCTCATGACGGGGATGCGGAGCCTCGCG
GCGGGGTCGCCATTGGCACTGATCTGGTTAACGGGGACCATCCGGCAGCTAAAGGGGTAGTGGA
AGGTGCTGGTGACGAGGACACAGGGGACGGAGGGAACAGTGGCCTGGCATCGACTGCTGATGTG
GTTGCTGAGGAGATGGCACCCTTTGAAGATGACTACGATGATGAGATGTTGGAGGAGCAGCTTG
TTGGAGATGTGATTCGTGCTTACAGTAATGGCAGAAACTTAGATTCAGATGGAGTGGATTGGGA
GGCAGAGGATGAGATGGAGTTCAATGACGATGCTGACAATAGTGATTTTATGGATGATGCTGAC
GATAGTGATTTTATGGATGATGCTTATGAAGGTGGCAATTCCAAACCAATTCAAAATCATGCTA
AGTTGGAAATACAAGACTGGGTGAACCAGAAAGTTGTTTTGAGTGGAGGGAGGTGTGAGGTGAG
GGGCGAGGGGGATCTGGAGGAAGAATTGGGTGTGGGAAAGGAAGCAGATGAGGAGGACGTGGAA
CCAAAGAGTGAAGCAGCTCCAGGTTCTGATAAAAGGGTCTTGCAGTTAGAAATCCTAGGTTCCG
ATGAGGAAATCAAGGTGCTTGAAAATATGAGTAGTGCCCCATCCAGGAAGGCGTCAGTTCAATC
GAAGTTACCAACTATTCCATCTTGTGTTGCATGGAGAACCCGATCATCATGGGGGGTAAATCAA
GATAGACTATCGTACGATACATATTTTGAGGAATTATCTGATGAGCCAAAAGAGGATGATGATG
ATACAGAGGTGGAACTTGATGAAGTTGAGGATGACAACAATGACGATGACAGTAGTGATGCTTA
TGATAAAGATGACGAAGAGAAGGAGGAAGAGGAAGAAGAGGCTGAAAGAAGAAAACTTAATAAC
AGGATTTGCACATCCGATGAAGACATGATCAATATTACTGTTCCTACATCAAGATATGATATGT
TTAAGAAAAAAAATTCCTCAAGATATGATATTGAGTGGGTGGAGGATGAAGATGCAAGTGTTGA
TATGTTACAGCCAGTTTCCTTTAAGAAAGATAGCAGCTGGAAGCCTGTGGCTGTTGGCAACGAC
ACATTTACTGAGCAACAAAAGCGATCACGATTTACTTGGGAGCTTGAGAGGAGGAAAAAGCTTA
AGCTTGAGATGAAGACAAATCCTTTGCATGAGCGGGATTTGGACTCAGATCCGAACTCATCAGG
TTCTGACCAGATCAGAAAGTATGGTTTCAAAAGTGATGGGAGTCATAAAGTTGATAGGAAAAAG
AAGCATACATCGCCCAAATCGGGCAAGAAACCCAGCAGCGCAATCATACTAAAGCGGCAGTCTC
TTTTGAAGCTTTTGGTAGATAAAATGAGTGGTGATAAAAGTTTAGCATCTTTTCCATTTGATCA
GAATCCTCAGCTTCAGTTTATTTTCAAAGAAATGCATCCATTGGTATTTTCATTTGGAGATGAA
GATCTAGTAGCAGCTGACAGGCCAGAGCAAGATGTTGGATTGGATATGTTATGGGCTGACTTTG
ACTTTGCTTTAGAGTCTGAGAATATCGGTACTTATTATGATGATGAGTGTCAAGAAGGCAATCA
ACTAGATTTTTCTCTTGCCTCAGTAACACCCTGTTCTCGTGGGAAGCATGAATTTGTTATTGAT
GATCAAATAGGGATCAGATGCAAATACTGTTCGTTGGTAAACCTGGAGATCAAATTCATGTTTC
CATCACTGGTGTCAGTGTTTGGCGAGAAATCAGCATGGCCAAATGACAAAGGCGTGAAGAATAC
ACTGATGTTTCATGATCTTTATGAACAAGGAGTCAATGACACTGAACAATCTCAAGATATTCAT

CAATATGGAACGGTGTGGAATCTTATTCCAGGGGTCATCAGTACTATGTATGAGCATCAGCGTG
AAGCATTTGAATTTATGTGGACAAATTTAGTTGGTGATATTAGACTTGATGAGATAAAGCATGG
AGCAAAACCTGATGTTGTTGGTGGATGTGTTATCTGTCATGCTCCTGGAACAGGAAAGACACGA
TTAGCTATTGTATTTATCCAGACATACATGAAAGTGTTTCCAGACTGTCGGCCAGTGATTATTG
CACCACGTGGTATGCTCTTTGCTTGGGATGAGGAATTTAAGAAATGGAATGTTGATGTTCCTTT
TCATATACTAAACACAACTGATTACACTGGAAAAGAGGACCGGGACATATGCAAGTTAATAAAG
AAAGAACATAGGACAGAAAAGTTGACAAGACTAGTCAAACTGCTTTCATGGAACAAAGGCCATG
GTATTCTTGGAATAAGTTATGGTCTGTACACAAAACTGACCTCTGAAAAACCTGGCTGCACAGA
AGAAAACAAAGTAAGAAGCATTCTTCTTGATAACCCTGGCTTACTTGTTCTTGATGAAGGACAT
ACACCTAGGAATGAGCGCAGTGTTATGTGGAAAACTCTAGGAAATGTGAAAACTGAGAAGCGTA
TAATTTTATCTGGAACTCCTTTTCAGAACAATTTTCTTGAGCTTTACAACATTCTTTGTCTGGT
AAGGCCTAGATTTGGTGAAATGTTTTTGACGAAGTCAAGAGTAGGTCGAAGACATTATGTCTCA
AAAAAGCAAAAGGATAAGTTTTCTGATAAATATGAAAAGGGTGTTTGGGCATCACTGACTAGCA
ATGTAACTGATGATAATGCGGAGAAAGTAAGATCAATATTGAAACCATTTGTTCATATACATAA
TGGCAATATTCTTCGAACTCTTCCAGGACTCAGGGAGAGTGTAATTATTTTGAAGCCTCTTCCC
CTTCAAAAGAGTATCATTAAAAAGGTGGAAAACATTGGTTCTGGTAACAACTTCGAACATGAAT
ATGTCATTTCTTTAGCTTCTACACACCCTTCCCTTGTAACCGCCATTAACATGTCTGAGGAGGA
AGCTTCACTTATTGATAAACCTATGCTTGCTAAAGTGAGATCAAATCCATATGAAGGGGTAAAA
ACAAGATTTGTGATCGAAGTTGTTCGTTTGTCTGAAGCATTAAGAGAGAAGGTTTTGATTTTTA
GCCAATTTATTCAGCCTCTAGAGTTGATTAAAGAGCATCTTCGCAAGTTCTTCAAATGGAGAGA
AGGGAAAGAAATTCTTCAAATGGATGGAAAGATCCTTCCAAGATATCGCCAGGCTTCCATTGAA
GCCTTCAATAATCCAAATAATGATTCCAGGGTGTTACTTGCATCTACAAGAGCATGCTGTGAAG
GGATTAGCTTGACAGGTGCTTCAAGAATTGTGCTTCTAGATGTTGTTTGGAACCCAGCTGTTGG
AAGGCAAGCCATCAGCAGAGCATTTAGGATAGGTCAGAAGAAATTTGTATATACATATAATTTG
ATAACTTATGGAACAGGTGAAGGTGACAAATATGATAGGCAAGCAGAAAAGGATCACTTATCCA
AGTTGGTCTTCTCTACAGAAGACGAGTTCAATAATGTTAGGAACATGTTATCTAAAGCTGAAAT
GGAGCACTGTTCTAAGTTTATCTCAGAAGATAAAGTTTTGGAGGAGATGACTTCCCACGATCAA
CTTAAAGGAATGTTTTTGAAGATCCATTATCCACCAACTGAGTCAAACATTGTCTATAGTTACA
ATCAAATTGCTACTGAGTGA

>Rmr1-A632 | genomic | 6393 bp
ATGGATCGCGCCACGCCGCGCGTTTGCGGCCGCCGCGGCGTATCCCAAGCGGCGGTGGAAGCTG
CGCCGTCCTCCTCCCGCGCGCGCCGCCGCGATAAGGCGCCGGCCGTTGTCATGGACCTTGGCGA
CGACGACTGCGGCGGTGGCGGGGCCAGGAAGACGGTTGGTGGCGCTGCAGGTAGGTGCGAGGGA
TCCACGAAGGCTCCTTTGCCTCTGCTGCCGCCGATGATGGTGCCGGCGGGAGCGGTGGCGCTGC
GGACACGATCGAGGAGGCGGGCGATGCTGGCGGCGGCAGTGGTGGAAGAGGCGCCAACGAAGAA
GAAGAAGAAGGAAGGAGCGATCCCAGATGCCGCGGAGGCGCCCCGTGGCCACGGGAGCAAGGCC
GCTGCTACCTCGATGGCGACGTCGAGCCATAAGCGGCGCGCTGGGACCTCGAGGTCGACGTCGA
GAGATAAGCGGCGCGCGCGCTCGGGACGTGCCTCGGAGCCAGCTCGCGTGGGCCGCGCGCGCAA
GCGCAAAAGGAACGAGTTGGAGGCCCCCGCTCGGAGAGAACGTGTGAAGGCGCCATGTGTCAGT
GAAAGTGATGACAACAGCGGGCGAGGCGATGACGCTTCTCATGACGGGGATGCGGAGCCTCGCG
TCGGGGTCGCCATTGGCACTGATCTGGTTAACGGGGATCATCCGGCAGCTAAAGGTGAGGATCA
TATTAAGAGAAGAGTGCTGTGAATTTGAATGATGACTTGGTCTCATGTAATGCAGAGGTAGTGG
AAGGTGCTGGTGACGAGGACACAGGGGACGGAGGGAACAGTGGCCTGGCATCGACTGCTGATGT
GTTTGCTGAGGAGATGGCACCCTTTGAAGATGACTACGATGATGAGATGTTGGAGGAGCAGCTT
GTTGGAGATGTGATTCGTGCTTACAGTAATGGCAGAAACTTCGATTCAGATGGAGTGGATTGGG

AGGCAGAGGATGAGATGGAGTTCAATGACGATGCTGACAATAGTGATTTTATGGATGATGCTGA
CGATAGTGATTTTATGGATGATGCTTATGAAGGTGGCAATTCCAAACCAATTCAAAATCATGCT
AAGTTGGAAATACAAGACTGGGTGAACCAGAAAGTTGTTTTGAGTGGAGGGAGGTGTGAGGCGA
GGGGCGAGGGGGATCTGGAGGAAGAATTGGATGTGGGAAAGGAAGCAGATGAGGAGGACGTGGA
ACCAAAGAGTGAAGCGGCTCCAGGTTCTGATAAAAGGGTCTTGCAGTTAGAAATCCTAGGTTCC
GATGAGGAAATCAAGGTGCTTGAAAATATGAGTAGTGCCCCATCCAGGAAGGCGTCAGTTCAAT
CGAAGTTACCAACTATTCCATCTTGTGTTGCATGGAGAACCCGATCATCATGGGGGGTAAATCA
AGATAGACTATCGTACGATACATATTTTGAGGAATTATCTGATGAGCCAAAAGAGGATGATGAT
GATACAGAGGTGGAACTTGATGAAGTTGAGGATGACAACAATGACGATGACAGTAGTGATGCTT
ATGATAAAGATGACGAAGAGAAGGAGGAAGAGGAAGAAGAGGCTGAAAGAAGAAAACTTAATAA
CAGGATTTGCACATCCGATGAAGACATGATCAATATTACTGTTCCTACATCAAGATATGATATG
TTTAAGAAAAAAAATTCCTCAAGATATGATATTGAGTGGGTGGAGGATGAAGATGCAAGTGTTG
ATATGTTACAGCCAGTTTCCTTTAAGAAAGATAGCAGCTGGAAGCCTGTGGCTGTTGGCAACGA
CACATTTACTGAGCAACAAAAGCGATCACGATTTACTTGGGAGCTTGAGAGGAGGAAAAAGCTT
AAGCTTGAGATGAAGACAAATCCTTTGCATGAGCGGGATTTGGACTCAGATCCGAACTCATCAG
GTTCTGACCAGATCAGAAAGTATGGTTTCAAAAGTGATGGGAGTCATAAAGTTGATAGGAAAAA
GAAGCATACATCGCCCAAATCGGGCAAGAAACCCAGCAGCGCAATCATACTAAAGCGGCAGTCT
CTTTTGAAGCTTTTGGTAGATAAAATGAGTGGTGATAAAAGTTTAGCATCTTTTCCATTTGATC
AGAATCCTCAGCTTCAGTTTATTTTCAAAGAAATGCATCCATTGGTATTTTCATTTGGAGATGA
AGATCTAGTAGCAGCTGACAGGCCAGAGCAAGATGTTGGATTGGATATGTTATGGGCTGACTTT
GACTTTGCTTTAGAGTCTGAGAATATCGGTACTTATTATGATGATGAGGTACATTCAGTACTGG
TTTTCATTTTATTTTTATCTATCAAAACACATGCAGGTATAAATATGTGTGGTTTATTTATTTG
AACACTTACTCTACAACTAGAACTATGGTAAAATTAACAATGTCCAACTGGAGATGCACTACCA
TGTTATTCTCAGCAAAAAGTTTGTCAATGGCGACAAACGCAAAAAGTTTGATGAAACAATCTCG
ATCAAACCAAAAAACTCTCATTATTCACCATCTGGCACAAGGGCAAAGATGCAGGAGATGCCCC
TTGCTCTGGCCAAACCCCAATCATGCAACTCCTCTCTAGGGAGCAGAACTCCAGCTATTTTTGG
AGATGCACCATCAAACACATATCAGCTTTGTCATTTCCAAATTGCCCATGCTCCAAGGGTGATG
ATCGAATTAAGCCCATTTTGTTCCTCACCATTTTTTTCAATGGACCTTGTGAAACCTAACCATC
AACCCTTGCTTCTCCCCTTGCCGATCATTCATCAAAGGAAACATCATCTATATTTGGAGGACCT
GCAGGCTGCAGCAGTCTAAACCAGAATTCTAATGCAAAAACACACAATGTGAGCAGGTGATTGA
TCATCTCAGCTTGATCACAAAGAGCACAGCAGGTAGGGTGCAGAAGACTCATCATGCATGCTGA
TCGGCAGTCCATTATCTATTATGAGTAAGTAACCACAAAAGAACCAACACTTCCTGGGGCCCAA
GATTTTCAAATTCTTTCCCAAGGCCCAAGAGGACAGAACCATGAAATAAACCCTTATCAACAGA
CTTGCCTCAATCATCTACATCTATCGTTAACATAACTAACAAGAGAAGAAATAAACCATTTGAG
CTCACTTTGATAGTACAACACTTCTGTGAACAATATGTGCATGTGAAATGTAGCCTCGTTTTCT
CATAATGCATATATCTGTGGTGTTTGTCAAGGGCCTCACACTGCTGTAGTGTCCATAAAGAAAG
CACAGGAATCAACAATCCTTCTTCGGGAACACTATTGAAGCTAGAACCCAGAAATGTACAGAAA
AAACTATTAGGGCATGGAAAGCATTCTTTCTGCATTTTCTTCAGTTTGATCTGGCTCTATTGTT
TCTCAGATCATGTCTACGTCATGGTAGTTTTTTTTCTCGAATGCGCAGGAGAGATGCGCATCAT
TATATTAAGAAGAGATGAAAAGGTCCAAAATAGACCAGCTCAAGATAGTACAAAAGGCCCTTTA
TGGCGGCCAAAAAGTAAGATACAGAAAATGATCCATTAAATCAATCCTACTATAAATCCGACCG
AAGAGGGGCAGCAAGGTAGTTTGCAGCTAATGTGCAGGAAACGTCGTTTCCCAGAATAGAGTAA
CTTTGTAAACATTAGAAATGTTTTTTTAGGTCAAGAAATCTAGTTCACTTAAAGGAGTTACTAT
AGAATAGTTTATAACCATAACTTTAAGGGGCAATTCAGATAGCCTCAGAAGGTCAAGAAAACTT
TATTATAGCCTGTGTGAATGTTTAGACAAAAAGGAAGCAGAAATGATTGTTCTTTGTCAAAAGT
TTCATCATCAGTGTGTTTCATTTAATGATTTCCTTTTGTCCCAACATTAATGACTTAGTTACAT

GTTGTTTTTGCGAATTAAATTTTCTAACTTGACGACGTTACTCAAACATATGTAGTATTCTGCA
TATCTGATACCACAGGTTGGTTCCTACTCTTAATTCGGCAATCAACATAAATAGTTTCATCTTT
TAGAAACTAGACACCCTTACTGCTTTCATGTAAAAGTTATATAGCATTTCAAGGTCGTGCGTCC
ATGATATACTACTCGATTTTTAATGTTTATTTTCTTAAATGCAAGAGTTTGTACCATTGTAGAT
ACTTTGATTTGCCACTGTGAGAATGACTAAATGAACTTATTAGCTTATGTTGTATTGTAGTGTC
AAGAAGGCAATCAACTAGATTTTTCTCTTGCCCCAGTAACACCCTGTTCTCGTGGGAAGCATGA
ATTTGTTATTGATGATCAAATAGGGATCAGATGCAAATACTGTTCCTTGGTAAACCTGGAGATC
AAATTCATGTTTCCATCACTGGTAAGCTTTATTATGGTCATAAATCATGACATCTATTCTACAG
TATAAGCTTTTAAATTTGGTCATTTAAGTCATCATTTTCTTTTAGGTGTCAGTGTTTGCCGAGA
AATCAGCATGGCCAAATGACAAAGGCGTGAAAAATACACTGATGTTTCATGATCTTTATGAACA
AGGAGTCAATGACACTGAACAATCTCAAGATATTCATCAATATGGAACGGTGTGGAATCTTATT
CCAGGGGTCATCAGTACTATGTATGAGCATCAGCGTGAAGCATTTGAATTTATGTGGACAAATT
TAGTTGGTGATATTAGACTTGATGAGATAAAGCATGGAGCAAAACCTGATGTTGTTGGTGGATG
TGTTATCTGTCATGCTCCTGGAACAGGAAAGACACGATTAGCTATTGTGTTTATCCAGACATAC
ATGAAGGTGTTTCCAGACTGTCGGCCAGTGATTATTGCACCACGTGGTATGCTCTTTGCTTGGG
ATGAGGAATTTAAGAAATGGAATGTTGATGTTCCTTTTCATATACTAAACACAACTGATTACAC
TGGAAAAGAGGATCGGGAGATATGCAAGTTAATAAAGAAAGAACATAGGACAGAAAAGTTGACA
AGACTAGTCAAACTGCTTTCATGGAACAAAGGTCATGGTATTCTTGGAATAAGTTATGGTCTGT
ACACAAAACTGACCTCTGAAAAACCTGGCTGCACAGAAGAAAACAAAGTAAGAAGCATTCTTCT
TGATAACCCTGGCTTACTTGTTCTTGATGAAGGACATACACCTAGGAATGAACGCAGTGTTATG
TGGAAAACTCTAGGAAATGTGAAAACTGAGAAGCGTATAATTTTATCCGGAACTCCTTTTCAGA
ACAATTTTCTTGAGCTTTACAACATTCTTTGTCTGGTAAGGCCTAGATTTGGTGAAATGTTTTT
GACGAAGTCAAGAGTAGGTCGAAGACATTATGTCTCAAAAAAGCAAAAGGATAAGTTTTCTGAT
AAATATGAAAAGGGTGTTTGGGCATCACTGACTAGCAATGTAACTGATGATAATGCGGAAAAAG
TAAGATCAATATTGAAACCATTTGTTCATATACATAATGGCAATATTCTTCGAACTCTTCCAGG
ACTCAGGGAGAGTGTAATTATTCTGAAGCCTCTTCCCCTTCAAAAGAGTATCATTAAAAAGGTG
GAAAACATTGGTTCTGGTAACAACTTCGAACATGAATATGTCATTTCTTTAGCTTCTACACACC
CTTCCCTTGTAACCGCCATTAACATGTCTGAGGAGGAAGCTTCACTTATTGATAAACCTATGCT
TGCTAAAGTGAGATCAAATCCATATGAAGGGGTAAAAACAAGATTTGTGATCGAAGTTGTTCGT
TTGTCTGAAGCATTAAGAGAGAAGGTTTTGATTTTTAGCCAATTTATTCAGCCTCTAGAGTTGA
TAAAAGAGCATCTTCGCAAGTTCTTCAAATGGAGAGAAGGGAAAGAAATTCTTCAAATGGATGG
AAAGATCCTTCCAAGATATCGCCAGGCTTCCATTGAAGCCTTCAATAATCCAAATAATGATTCC
AGGGTGTTACTTGCATCTACAAGAGCATGCTGTGAAGGGATTAGCTTGACAGGTGCTTCAAGAA
TTGTGCTTCTAGATGTTGTTTGGAACCCAGCTGTTGGAAGGCAAGCCATCAGCAGAGCATTTAG
GATAGGTCAGAAGAAATTTGTATATACATATAATTTGATAACTTATGGAACAGGTGAAGGTGAC
AAATATGATAGGCAAGCAGAAAAGGATCACTTATCCAAGTTGGTCTTCTCTACAGAAGACGAGT
TCAATAATGTTAGGAACATGTTATCTAAAGCTGAAATGGAGCACTGTTCTAAGTTTATCTCAGA
AGATAAAGTTTTGGAGGAGATGACTTCCCACGATCAACTTAAAGGAATGTTTTTGAAGATCCAT
TATCCACCAACTGAGTCAAACATTGTCTATAGTTACAATCAAATTGCTACTGAGTGA

>Rmr1-A632 | coding sequence | 4308 bp
ATGGATCGCGCCACGCCGCGCGTTTGCGGCCGCCGCGGCGTATCCCAAGCGGCGGTGGAAGCTG
CGCCGTCCTCCTCCCGCGCGCGCCGCCGCGATAAGGCGCCGGCCGTTGTCATGGACCTTGGCGA
CGACGACTGCGGCGGTGGCGGGGCCAGGAAGACGGTTGGTGGCGCTGCAGGTAGGTGCGAGGGA
TCCACGAAGGCTCCTTTGCCTCTGCTGCCGCCGATGATGGTGCCGGCGGGAGCGGTGGCGCTGC
GGACACGATCGAGGAGGCGGGCGATGCTGGCGGCGGCAGTGGTGGAAGAGGCGCCAACGAAGAA

GAAGAAGAAGGAAGGAGCGATCCCAGATGCCGCGGAGGCGCCCCGTGGCCACGGGAGCAAGGCC
GCTGCTACCTCGATGGCGACGTCGAGCCATAAGCGGCGCGCTGGGACCTCGAGGTCGACGTCGA
GAGATAAGCGGCGCGCGCGCTCGGGACGTGCCTCGGAGCCAGCTCGCGTGGGCCGCGCGCGCAA
GCGCAAAAGGAACGAGTTGGAGGCCCCCGCTCGGAGAGAACGTGTGAAGGCGCCATGTGTCAGT
GAAAGTGATGACAACAGCGGGCGAGGCGATGACGCTTCTCATGACGGGGATGCGGAGCCTCGCG
TCGGGGTCGCCATTGGCACTGATCTGGTTAACGGGGATCATCCGGCAGCTAAAGGGGTAGTGGA
AGGTGCTGGTGACGAGGACACAGGGGACGGAGGGAACAGTGGCCTGGCATCGACTGCTGATGTG
TTTGCTGAGGAGATGGCACCCTTTGAAGATGACTACGATGATGAGATGTTGGAGGAGCAGCTTG
TTGGAGATGTGATTCGTGCTTACAGTAATGGCAGAAACTTCGATTCAGATGGAGTGGATTGGGA
GGCAGAGGATGAGATGGAGTTCAATGACGATGCTGACAATAGTGATTTTATGGATGATGCTGAC
GATAGTGATTTTATGGATGATGCTTATGAAGGTGGCAATTCCAAACCAATTCAAAATCATGCTA
AGTTGGAAATACAAGACTGGGTGAACCAGAAAGTTGTTTTGAGTGGAGGGAGGTGTGAGGCGAG
GGGCGAGGGGGATCTGGAGGAAGAATTGGATGTGGGAAAGGAAGCAGATGAGGAGGACGTGGAA
CCAAAGAGTGAAGCGGCTCCAGGTTCTGATAAAAGGGTCTTGCAGTTAGAAATCCTAGGTTCCG
ATGAGGAAATCAAGGTGCTTGAAAATATGAGTAGTGCCCCATCCAGGAAGGCGTCAGTTCAATC
GAAGTTACCAACTATTCCATCTTGTGTTGCATGGAGAACCCGATCATCATGGGGGTAAATCAA
GATAGACTATCGTACGATACATATTTTGAGGAATTATCTGATGAGCCAAAAGAGGATGATGATG
ATACAGAGGTGGAACTTGATGAAGTTGAGGATGACAACAATGACGATGACAGTAGTGATGCTTA
TGATAAAGATGACGAAGAGAAGGAGGAAGAGGAAGAAGAGGCTGAAAGAAGAAAACTTAATAAC
AGGATTTGCACATCCGATGAAGACATGATCAATATTACTGTTCCTACATCAAGATATGATATGT
TTAAGAAAAAAAATTCCTCAAGATATGATATTGAGTGGGTGGAGGATGAAGATGCAAGTGTTGA
TATGTTACAGCCAGTTTCCTTTAAGAAAGATAGCAGCTGGAAGCCTGTGGCTGTTGGCAACGAC
ACATTTACTGAGCAACAAAAGCGATCACGATTTACTTGGGAGCTTGAGAGGAGGAAAAAGCTTA
AGCTTGAGATGAAGACAAATCCTTTGCATGAGCGGGATTTGGACTCAGATCCGAACTCATCAGG
TTCTGACCAGATCAGAAAGTATGGTTTCAAAAGTGATGGGAGTCATAAAGTTGATAGGAAAAAG
AAGCATACATCGCCCAAATCGGGCAAGAAACCCAGCAGCGCAATCATACTAAAGCGGCAGTCTC
TTTTGAAGCTTTTGGTAGATAAAATGAGTGGTGATAAAAGTTTAGCATCTTTTCCATTTGATCA
GAATCCTCAGCTTCAGTTTATTTTCAAAGAAATGCATCCATTGGTATTTTCATTTGGAGATGAA
GATCTAGTAGCAGCTGACAGGCCAGAGCAAGATGTTGGATTGGATATGTTATGGGCTGACTTTG
ACTTTGCTTTAGAGTCTGAGAATATCGGTACTTATTATGATGATGAGTGTCAAGAAGGCAATCA
ACTAGATTTTTCTCTTGCCCCAGTAACACCCTGTTCTCGTGGGAAGCATGAATTTGTTATTGAT
GATCAAATAGGGATCAGATGCAAATACTGTTCCTTGGTAAACCTGGAGATCAAATTCATGTTTC
CATCACTGGTGTCAGTGTTTGCCGAGAAATCAGCATGGCCAAATGACAAAGGCGTGAAAAATAC
ACTGATGTTTCATGATCTTTATGAACAAGGAGTCAATGACACTGAACAATCTCAAGATATTCAT
CAATATGGAACGGTGTGGAATCTTATTCCAGGGGTCATCAGTACTATGTATGAGCATCAGCGTG
AAGCATTTGAATTTATGTGGACAAATTTAGTTGGTGATATTAGACTTGATGAGATAAAGCATGG
AGCAAAACCTGATGTTGTTGGTGGATGTGTTATCTGTCATGCTCCTGGAACAGGAAAGACACGA
TTAGCTATTGTGTTTATCCAGACATACATGAAGGTGTTTCCAGACTGTCGGCCAGTGATTATTG
CACCACGTGGTATGCTCTTTGCTTGGGATGAGGAATTTAAGAAATGGAATGTTGATGTTCCTTT
TCATATACTAAACACAACTGATTACACTGGAAAAGAGGATCGGGAGATATGCAAGTTAATAAAG
AAAGAACATAGGACAGAAAAGTTGACAAGACTAGTCAAACTGCTTTCATGGAACAAAGGTCATG
GTATTCTTGGAATAAGTTATGGTCTGTACACAAAACTGACCTCTGAAAAACCTGGCTGCACAGA
AGAAAACAAAGTAAGAAGCATTCTTCTTGATAACCCTGGCTTACTTGTTCTTGATGAAGGACAT
ACACCTAGGAATGAACGCAGTGTTATGTGGAAAACTCTAGGAAATGTGAAAACTGAGAAGCGTA
TAATTTTATCCGGAACTCCTTTTCAGAACAATTTTCTTGAGCTTTACAACATTCTTTGTCTGGT
AAGGCCTAGATTTGGTGAAATGTTTTTGACGAAGTCAAGAGTAGGTCGAAGACATTATGTCTCA

AAAAAGCAAAAGGATAAGTTTTCTGATAAATATGAAAAGGGTGTTTGGGCATCACTGACTAGCA
ATGTAACTGATGATAATGCGGAAAAAGTAAGATCAATATTGAAACCATTTGTTCATATACATAA
TGGCAATATTCTTCGAACTCTTCCAGGACTCAGGGAGAGTGTAATTATTCTGAAGCCTCTTCCC
CTTCAAAAGAGTATCATTAAAAAGGTGGAAAACATTGGTTCTGGTAACAACTTCGAACATGAAT
ATGTCATTTCTTTAGCTTCTACACACCCTTCCCTTGTAACCGCCATTAACATGTCTGAGGAGGA
AGCTTCACTTATTGATAAACCTATGCTTGCTAAAGTGAGATCAAATCCATATGAAGGGGTAAAA
ACAAGATTTGTGATCGAAGTTGTTCGTTTGTCTGAAGCATTAAGAGAGAAGGTTTTGATTTTTA
GCCAATTTATTCAGCCTCTAGAGTTGATAAAAGAGCATCTTCGCAAGTTCTTCAAATGGAGAGA
AGGGAAAGAAATTCTTCAAATGGATGGAAAGATCCTTCCAAGATATCGCCAGGCTTCCATTGAA
GCCTTCAATAATCCAAATAATGATTCCAGGGTGTTACTTGCATCTACAAGAGCATGCTGTGAAG
GGATTAGCTTGACAGGTGCTTCAAGAATTGTGCTTCTAGATGTTGTTTGGAACCCAGCTGTTGG
AAGGCAAGCCATCAGCAGAGCATTTAGGATAGGTCAGAAGAAATTTGTATATACATATAATTTG
ATAACTTATGGAACAGGTGAAGGTGACAAATATGATAGGCAAGCAGAAAAGGATCACTTATCCA
AGTTGGTCTTCTCTACAGAAGACGAGTTCAATAATGTTAGGAACATGTTATCTAAAGCTGAAAT
GGAGCACTGTTCTAAGTTTATCTCAGAAGATAAAGTTTTGGAGGAGATGACTTCCCACGATCAA
CTTAAAGGAATGTTTTTGAAGATCCATTATCCACCAACTGAGTCAAACATTGTCTATAGTTACA
ATCAAATTGCTACTGAGTGA

>rmr1-1 | coding sequence | 4308 bp
ATGGATCGCGCCACGCCGCGCGTTTGCGGCCGCCGCGGCGTATCCCAAGCGGCGGTGGAAGCTG
CGCCGTCCTCCTCCCGCGCGCGCCGCCGCGATAAGGCGCCGGCCGTTGTCATGGACCTTGGCGA
CGACGACTGCGGCGGTGGCGGGGCCAGGAAGACGGTTGGTGGCGCTGCAGGTAGGTGCGAGGGA
TCCACGAAGGCTCCTTCGCCTATGCTGCCGCCGATGATGGTGCCGGCGGGAGCGGTGGCGCTGC
GGACACGATCGAGGAGGCGGGCGATGCTGGCGGCGGCAGTGGTGGAAGAGGCGCCAACGAAGAA
GAAGAAGAAGGAAGGAGCGATCCCAGATGCCGCGGAGGCAACCCGTGGCCACGGGAGCAAGGCC
GCTGCGACCTCGATGGCGACGTCGAGCCATAAGCGGCGCGCTGGGACCTCGAGGTCGACGTCGA
GAGATAAGCGGCGCGCGCGCTCGGGACGTGCCTCGGAGCCAGCTCGCGTGGGCCGCGCGCGCAA
GCGCAAAAGGAACGAGTTGGAGGCCCCCGCTCGGAGAGAACGTGTGAAGGCGCCATGTGTCAGT
GAAAGTGATGACAACAGCGGGCGAGGCGATGACGCTTCTCATGACGGGGATGCGGAGCCTCGCG
GCGGGGTCGCCATTGGCACTGATCTGGTTAACGGGGACCATCCGGCAGCTAAAGGGGTAGTGGA
AGGTGCTGGTGACGAGGACACAGGGGACGGAGGGAACAGTGGCCTGGCATCGACTGCTGATGTG
GTTGCTGAGGAGATGGCACCCTTTGAAGATGACTACGATGATGAGATGTTGGAGGAGCAGCTTG
TTGGAGATGTGATTCGTGCTTACAGTAATGGCAGAAACTTAGATTCAGATGGAGTGGATTGGGA
GGCAGAGGATGAGATGGAGTTCAATGACGATGCTGACAATAGTGATTTTATGGATGATGCTGAC
GATAGTGATTTTATGGATGATGCTTATGAAGGTGGCAATTCCAAACCAATTCAAAATCATGCTA
AGTTGGAAATACAAGACTGGGTGAACCAGAAAGTTGTTTTGAGTGGAGGGAGGTGTGAGGTGAG
GGGCGAGGGGGATCTGGAGGAAGAATTGGGTGTGGGAAAGGAAGCAGATGAGGAGGACGTGGAA
CCAAAGAGTGAAGCAGCTCCAGGTTCTGATAAAAGGGTCTTGCAGTTAGAAATCCTAGGTTCCG
ATGAGGAAATCAAGGTGCTTGAAAATATGAGTAGTGCCCCATCCAGGAAGGCGTCAGTTCAATC
GAAGTTACCAACTATTCCATCTTGTGTTGCATGGAGAACCCGATCATCATGGGGGGTAAATCAA
GATAGACTATCGTACGATACATATTTTGAGGAATTATCTGATGAGCCAAAAGAGGATGATGATG
ATACAGAGGTGGAACTTGATGAAGTTGAGGATGACAACAATGACGATGACAGTAGTGATGCTTA
TGATAAAGATGACGAAGAGAAGGAGGAAGAGGAAGAAGAGGCTGAAAGAAGAAAACTTAATAAC
AGGATTTGCACATCCGATGAAGACATGATCAATATTACTGTTCCTACATCAAGATATGATATGT
TTAAGAAAAAAAATTCCTCAAGATATGATATTGAGTGGGTGGAGGATGAAGATGCAAGTGTTGA
TATGTTACAGCCAGTTTCCTTTAAGAAAGATAGCAGCTGGAAGCCTGTGGCTGTTGGCAACGAC

ACATTTACTGAGCAACAAAAGCGATCACGATTTACTTGGGAGCTTGAGAGGAGGAAAAAGCTTA
AGCTTGAGATGAAGACAAATCCTTTGCATGAGCGGGATTTGGACTCAGATCCGAACTCATCAGG
TTCTGACCAGATCAGAAAGTATGGTTTCAAAAGTGATGGGAGTCATAAAGTTGATAGGAAAAAG
AAGCATACATCGCCCAAATCGGGCAAGAAACCCAGCAGCGCAATCATACTAAAGCGGCAGTCTC
TTTTGAAGCTTTTGGTAGATAAAATGAGTGGTGATAAAAGTTTAGCATCTTTTCCATTTGATCA
GAATCCTCAGCTTCAGTTTATTTTCAAAGAAATGCATCCATTGGTATTTTCATTTGGAGATGAA
GATCTAGTAGCAGCTGACAGGCCAGAGCAAGATGTTGGATTGGATATGTTATGGGCTGACTTTG
ACTTTGCTTTAGAGTCTGAGAATATCGGTACTTATTATGATGATGAGTGTCAAGAAGGCAATCA
ACTAGATTTTTCTCTTGCCTCAGTAACACCCTGTTCTCGTGGGAAGCATGAATTTGTTATTGAT
GATCAAATAGGGATCAGATGCAAATACTGTTCGTTGGTAAACCTGGAGATCAAATTCATGTTTC
CATCACTGGTGTCAGTGTTTGGCGAGAAATCAGCATGGCCAAATGACAAAGGCGTGAAGAATAC
ACTGATGTTTCATGATCTTTATGAACAAGGAGTCAATGACACTGAACAATCTCAAGATATTCAT
CAATATGGAACGGTGTGGAATCTTATTCCAGGGGTCATCAGTACTATGTATGAGCATCAGCGTG
AAGCATTTGAATTTATGTGGACAAATTTAGTTGGTGATATTAGACTTGATGAGATAAAGCATGG
AGCAAAACCTGATGTTGTTGGTGGATGTGTTATCTGTCATGCTCCTGGAACAGGAAAGACACGA
TTAGCTATTGTATTTATCCAGACATACATGAAAGTGTTTCCAGACTGTCGGCCAGTGATTATTG
CACCACGTGGTATGCTCTTTGCTTGGGATGAGGAATTTAAGAAATGGAATGTTGATGTTCCTTT
TCATATACTAAACACAACTGATTACACTGGAAAAGAGGACCGGGACATATGCAAGTTAATAAAG
AAAGAACATAGGACAGAAAAGTTGACAAGACTAGTCAAACTGCTTTCATGGAACAAAGGCCATG
GTATTCTTGGAATAAGTTATGGTCTGTACACAAAACTGACCTCTGAAAAACCTGGCTGCACAGA
AGAAAACAAAGTAAGAAGCATTCTTCTTGATAACCCTGGCTTACTTGTTCTTGATGAAGGACAT
ACACCTAGGAATGAGCGCAGTGTTATGTGGAAAACTCTAGGAAATGTGAAAACTGAGAAGCGTA
TAATTTTATCTGGAACTCCTTTTCAGAACAATTTTCTTGAGCTTTACAACATTCTTTGTCTGGT
AAGGCCTAGATTTGGTGAAATGTTTTTGACGAAGTCAAGAGTAGGTCGAAGACATTATGTCTCA
AAAAAGCAAAAGGATAAGTTTTCTGATAAATATGAAAAGGGTGTTTGGGCATCACTGACTAGCA
ATGTAACTGATGATAATGCGGAGAAAGTAAGATCAATATTGAAACCATTTGTTCATATACATAA
TGGCAATATTCTTCGAACTCTTCCAGGACTCAGGGAGAGTGTAATTATTTTGAAGCCTCTTCCC
CTTCAAAAGAGTATCATTAAAAAGGTGGAAAACATTGGTTCTGGTAACAACTTCGAACATGAAT
ATGTCATTTCTTTAGCTTCTACACACCCTTCCCTTGTAACCGCCATTAACATGTCTGAGGAGGA
AGCTTCACTTATTGATAAACCTATGCTTGCTAAAGTGAGATCAAATCCATATGAAGGGGTAAAA
ACAAGATTTGTGATCGAAGTTGTTCGTTTGTCTGAAGCATTAAGAGAGAAGGTTTTGATTTTTA
GCCAATTTATTCAGCCTCTAGAGTTGATTAAAGAGCATCTTCGCAAGTTCTTCAAATGGAGAGA
AGGGAAAGAAATTCTTCAAATGGATGGAAAGATCCTTCCAAGATATCGCCAGGCTTCCATTGAA
GCCTTCAATAATCCAAATAATGATTCCAGGGTGTTACTTGCATCTACAAGAGCATGCTGTGAAG
GGATTAGCTTGACAGGTGCTTCAAGAATTGTGCTTCTAGATGTTGTTTGGAACCCAGTTGTTGG
AAGGCAAGCCATCAGCAGAGCATTTAGGATAGGTCAGAAGAAATTTGTATATACATATAATTTG
ATAACTTATGGAACAGGTGAAGGTGACAAATATGATAGGCAAGCAGAAAAGGATCACTTATCCA
AGTTGGTCTTCTCTACAGAAGACGAGTTCAATAATGTTAGGAACATGTTATCTAAAGCTGAAAT
GGAGCACTGTTCTAAGTTTATCTCAGAAGATAAAGTTTTGGAGGAGATGACTTCCCACGATCAA
CTTAAAGGAATGTTTTTGAAGATCCATTATCCACCAACTGAGTCAAACATTGTCTATAGTTACA
ATCAAATTGCTACTGAGTGA

>rmr1-2 | coding sequence | 4308 bp
ATGGATCGCGCCACGCCGCGCGTTTGCGGCCGCCGCGGCGTATCCCAAGCGGCGGTGGAAGCTG
CGCCGTCCTCCTCCCGCGCGCGCCGCCGCGATAAGGCGCCGGCCGTTGTCATGGACCTTGGCGA
CGACGACTGCGGCGGTGGCGGGGCCAGGAAGACGGTTGGTGGCGCTGCAGGTAGGTGCGAGGGA

TCCACGAAGGCTCCTTCGCCTATGCTGCCGCCGATGATGGTGCCGGCGGGAGCGGTGGCGCTGC
GGACACGATCGAGGAGGCGGGCGATGCTGGCGGCGGCAGTGGTGGAAGAGGCGCCAACGAAGAA
GAAGAAGAAGGAAGGAGCGATCCCAGATGCCGCGGAGGCAACCCGTGGCCACGGGAGCAAGGCC
GCTGCGACCTCGATGGCGACGTCGAGCCATAAGCGGCGCGCTGGGACCTCGAGGTCGACGTCGA
GAGATAAGCGGCGCGCGCGCTCGGGACGTGCCTCGGAGCCAGCTCGCGTGGGCCGCGCGCGCAA
GCGCAAAAGGAACGAGTTGGAGGCCCCCGCTCGGAGAGAACGTGTGAAGGCGCCATGTGTCAGT
GAAAGTGATGACAACAGCGGGCGAGGCGATGACGCTTCTCATGACGGGGATGCGGAGCCTCGCG
GCGGGGTCGCCATTGGCACTGATCTGGTTAACGGGGACCATCCGGCAGCTAAAGGGGTAGTGGA
AGGTGCTGGTGACGAGGACACAGGGGACGGAGGGAACAGTGGCCTGGCATCGACTGCTGATGTG
GTTGCTGAGGAGATGGCACCCTTTGAAGATGACTACGATGATGAGATGTTGGAGGAGCAGCTTG
TTGGAGATGTGATTCGTGCTTACAGTAATGGCAGAAACTTAGATTCAGATGGAGTGGATTGGGA
GGCAGAGGATGAGATGGAGTTCAATGACGATGCTGACAATAGTGATTTTATGGATGATGCTGAC
GATAGTGATTTTATGGATGATGCTTATGAAGGTGGCAATTCCAAACCAATTCAAAATCATGCTA
AGTTGGAAATACAAGACTGGGTGAACCAGAAAGTTGTTTTGAGTGGAGGGAGGTGTGAGGTGAG
GGGCGAGGGGGATCTGGAGGAAGAATTGGGTGTGGGAAAGGAAGCAGATGAGGAGGACGTGGAA
CCAAAGAGTGAAGCAGCTCCAGGTTCTGATAAAAGGGTCTTGCAGTTAGAAATCCTAGGTTCCG
ATGAGGAAATCAAGGTGCTTGAAAATATGAGTAGTGCCCCATCCAGGAAGGCGTCAGTTCAATC
GAAGTTACCAACTATTCCATCTTGTGTTGCATGGAGAACCCGATCATCATGGGGGGTAAATCAA
GATAGACTATCGTACGATACATATTTTGAGGAATTATCTGATGAGCCAAAAGAGGATGATGATG
ATACAGAGGTGGAACTTGATGAAGTTGAGGATGACAACAATGACGATGACAGTAGTGATGCTTA
TGATAAAGATGACGAAGAGAAGGAGGAAGAGGAAGAAGAGGCTGAAAGAAGAAAACTTAATAAC
AGGATTTGCACATCCGATGAAGACATGATCAATATTACTGTTCCTACATCAAGATATGATATGT
TTAAGAAAAAAAATTCCTCAAGATATGATATTGAGTGGGTGGAGGATGAAGATGCAAGTGTTGA
TATGTTACAGCCAGTTTCCTTTAAGAAAGATAGCAGCTGGAAGCCTGTGGCTGTTGGCAACGAC
ACATTTACTGAGCAACAAAAGCGATCACGATTTACTTGGGAGCTTGAGAGGAGGAAAAAGCTTA
AGCTTGAGATGAAGACAAATCCTTTGCATGAGCGGGATTTGGACTCAGATCCGAACTCATCAGG
TTCTGACCAGATCAGAAAGTATGGTTTCAAAAGTGATGGGAGTCATAAAGTTGATAGGAAAAAG
AAGCATACATCGCCCAAATCGGGCAAGAAACCCAGCAGCGCAATCATACTAAAGCGGCAGTCTC
TTTTGAAGCTTTTGGTAGATAAAATGAGTGGTGATAAAAGTTTAGCATCTTTTCCATTTGATCA
GAATCCTCAGCTTCAGTTTATTTTCAAAGAAATGCATCCATTGGTATTTTCATTTGGAGATGAA
GATCTAGTAGCAGCTGACAGGCCAGAGCAAGATGTTGGATTGGATATGTTATGGGCTGACTTTG
ACTTTGCTTTAGAGTCTGAGAATATCGGTACTTATTATGATGATGAGTGTCAAGAAGGCAATCA
ACTAGATTTTTCTCTTGCCTCAGTAACACCCTGTTCTCGTGGGAAGCATGAATTTGTTATTGAT
GATCAAATAGGGATCAGATGCAAATACTGTTCGTTGGTAAACCTGGAGATCAAATTCATGTTTC
CATCACTGGTGTCAGTGTTTGGCGAGAAATCAGCATGGCCAAATGACAAAGGCGTGAAGAATAC
ACTGATGTTTCATGATCTTTATGAACAAGGAGTCAATGACACTGAACAATCTCAAGATATTCAT
CAATATGGAACGGTGTGGAATCTTATTCCAGGGGTCATCAGTACTATGTATGAGCATCAGCGTG
AAGCATTTGAATTTATGTGGACAAATTTAGTTGGTGATATTAGACTTGATGAGATAAAGCATGG
AGCAAAACCTGATGTTGTTGGTGGATGTGTTATCTGTCATGCTCCTGGAACAGGAAAGACACGA
TTAGCTATTGTATTTATCCAGACATACATGAAAGTGTTTCCAGACTGTCGGCCAGTGATTATTG
CACCACGTGGTATGCTCTTTGCTTGGGATGAGGAATTTAAGAAATGGAATGTTGATGTTCCTTT
TCATATACTAAACACAACTGATTACACTGGAAAAGAGGACCGGGACATATGCAAGTTAATAAAG
AAAGAACATAGGACAGAAAAGTTGACAAGACTAGTCAAACTGCTTTCATGGAACAAAGGCCATG
GTATTCTTGGAATAAGTTATGGTCTGTACACAAAACTGACCTCTGAAAAACCTGGCTGCACAGA
AGAAAACAAAGTAAGAAGCATTCTTCTTGATAACCCTGGCTTACTTGTTCTTGATGAAGGACAT
ACACCTAGGAATGAGCGCAATGTTATGTGGAAAACTCTAGGAAATGTGAAAACTGAGAAGCGTA

TAATTTTATCTGGAACTCCTTTTCAGAACAATTTTCTTGAGCTTTACAACATTCTTTGTCTGGT
AAGGCCTAGATTTGGTGAAATGTTTTTGACGAAGTCAAGAGTAGGTCGAAGACATTATGTCTCA
AAAAAGCAAAAGGATAAGTTTTCTGATAAATATGAAAAGGGTGTTTGGCATCACTGACTAGCA
ATGTAACTGATGATAATGCGGAGAAAGTAAGATCAATATTGAAACCATTTGTTCATATACATAA
TGGCAATATTCTTCGAACTCTTCCAGGACTCAGGGAGAGTGTAATTATTTTGAAGCCTCTTCCC
CTTCAAAAGAGTATCATTAAAAAGGTGGAAAACATTGGTTCTGGTAACAACTTCGAACATGAAT
ATGTCATTTCTTTAGCTTCTACACACCCTTCCCTTGTAACCGCCATTAACATGTCTGAGGAGGA
AGCTTCACTTATTGATAAACCTATGCTTGCTAAAGTGAGATCAAATCCATATGAAGGGGTAAAA
ACAAGATTTGTGATCGAAGTTGTTCGTTTGTCTGAAGCATTAAGAGAGAAGGTTTTGATTTTTA
GCCAATTTATTCAGCCTCTAGAGTTGATTAAAGAGCATCTTCGCAAGTTCTTCAAATGGAGAGA
AGGGAAAGAAATTCTTCAAATGGATGGAAAGATCCTTCCAAGATATCGCCAGGCTTCCATTGAA
GCCTTCAATAATCCAAATAATGATTCCAGGGTGTTACTTGCATCTACAAGAGCATGCTGTGAAG
GGATTAGCTTGACAGGTGCTTCAAGAATTGTGCTTCTAGATGTTGTTTGGAACCCAGCTGTTGG
AAGGCAAGCCATCAGCAGAGCATTTAGGATAGGTCAGAAGAAATTTGTATATACATATAATTTG
ATAACTTATGGAACAGGTGAAGGTGACAAATATGATAGGCAAGCAGAAAAGGATCACTTATCCA
AGTTGGTCTTCTCTACAGAAGACGAGTTCAATAATGTTAGGAACATGTTATCTAAAGCTGAAAT
GGAGCACTGTTCTAAGTTTATCTCAGAAGATAAAGTTTTGGAGGAGATGACTTCCCACGATCAA
CTTAAAGGAATGTTTTTGAAGATCCATTATCCACCAACTGAGTCAAACATTGTCTATAGTTACA
ATCAAATTGCTACTGAGTGA

>rmr1-3 | coding sequence | 4308 bp
ATGGATCGCGCCACGCCGCGCGTTTGCGGCCGCCGCGGCGTATCCCAAGCGGCGGTGGAAGCTG
CGCCGTCCTCCTCCCGCGCGCGCCGCCGCGATAAGGCGCCGGCCGTTGTCATGGACCTTGGCGA
CGACGACTGCGGCGGTGGCGGGGCCAGGAAGACGGTTGGTGGCGCTGCAGGTAGGTGCGAGGGA
TCCACGAAGGCTCCTTCGCCTATGCTGCCGCCGATGATGGTGCCGGCGGGAGCGGTGGCGCTGC
GGACACGATCGAGGAGGCGGGCGATGCTGGCGGCGGCAGTGGTGGAAGAGGCGCCAACGAAGAA
GAAGAAGAAGGAAGGAGCGATCCCAGATGCCGCGGAGGCAACCCGTGGCCACGGGAGCAAGGCC
GCTGCGACCTCGATGGCGACGTCGAGCCATAAGCGGCGCGCTGGGACCTCGAGGTCGACGTCGA
GAGATAAGCGGCGCGCGCGCTCGGGACGTGCCTCGGAGCCAGCTCGCGTGGGCCGCGCGCGCAA
GCGCAAAAGGAACGAGTTGGAGGCCCCCGCTCGGAGAGAACGTGTGAAGGCGCCATGTGTCAGT
GAAAGTGATGACAACAGCGGGCGAGGCGATGACGCTTCTCATGACGGGGATGCGGAGCCTCGCG
GCGGGGTCGCCATTGGCACTGATCTGGTTAACGGGGACCATCCGGCAGCTAAAGGGGTAGTGGA
AGGTGCTGGTGACGAGGACACAGGGGACGGAGGGAACAGTGGCCTGGCATCGACTGCTGATGTG
GTTGCTGAGGAGATGGCACCCTTTGAAGATGACTACGATGATGAGATGTTGGAGGAGCAGCTTG
TTGGAGATGTGATTCGTGCTTACAGTAATGGCAGAAACTTAGATTCAGATGGAGTGGATTGGGA
GGCAGAGGATGAGATGGAGTTCAATGACGATGCTGACAATAGTGATTTTATGGATGATGCTGAC
GATAGTGATTTTATGGATGATGCTTATGAAGGTGGCAATTCCAAACCAATTCAAAATCATGCTA
AGTTGGAAATACAAGACTGGGTGAACCAGAAAGTTGTTTTGAGTGGAGGGAGGTGTGAGGTGAG
GGGCGAGGGGGATCTGGAGGAAGAATTGGGTGTGGGAAAGGAAGCAGATGAGGAGGACGTGGAA
CCAAAGAGTGAAGCAGCTCCAGGTTCTGATAAAAGGGTCTTGCAGTTAGAAATCCTAGGTTCCG
ATGAGGAAATCAAGGTGCTTGAAAATATGAGTAGTGCCCCATCCAGGAAGGCGTCAGTTCAATC
GAAGTTACCAACTATTCCATCTTGTGTTGCATGGAGAACCTGATCATCATGGGGGGTAAATCAA
GATAGACTATCGTACGATACATATTTTGAGGAATTATCTGATGAGCCAAAAGAGGATGATGATG
ATACAGAGGTGGAACTTGATGAAGTTGAGGATGACAACAATGACGATGACAGTAGTGATGCTTA
TGATAAAGATGACGAAGAGAAGGAGGAAGAGGAAGAAGAGGCTGAAAGAAGAAAACTTAATAAC
AGGATTTGCACATCCGATGAAGACATGATCAATATTACTGTTCCTACATCAAGATATGATATGT

TTAAGAAAAAAAATTCCTCAAGATATGATATTGAGTGGGTGGAGGATGAAGATGCAAGTGTTGA
TATGTTACAGCCAGTTTCCTTTAAGAAAGATAGCAGCTGGAAGCCTGTGGCTGTTGGCAACGAC
ACATTTACTGAGCAACAAAAGCGATCACGATTTACTTGGGAGCTTGAGAGGAGGAAAAAGCTTA
AGCTTGAGATGAAGACAAATCCTTTGCATGAGCGGGATTTGGACTCAGATCCGAACTCATCAGG
TTCTGACCAGATCAGAAAGTATGGTTTCAAAAGTGATGGGAGTCATAAAGTTGATAGGAAAAAG
AAGCATACATCGCCCAAATCGGGCAAGAAACCCAGCAGCGCAATCATACTAAAGCGGCAGTCTC
TTTTGAAGCTTTTGGTAGATAAAATGAGTGGTGATAAAAGTTTAGCATCTTTTCCATTTGATCA
GAATCCTCAGCTTCAGTTTATTTTCAAAGAAATGCATCCATTGGTATTTTCATTTGGAGATGAA
GATCTAGTAGCAGCTGACAGGCCAGAGCAAGATGTTGGATTGGATATGTTATGGGCTGACTTTG
ACTTTGCTTTAGAGTCTGAGAATATCGGTACTTATTATGATGATGAGTGTCAAGAAGGCAATCA
ACTAGATTTTTCTCTTGCCTCAGTAACACCCTGTTCTCGTGGGAAGCATGAATTTGTTATTGAT
GATCAAATAGGGATCAGATGCAAATACTGTTCGTTGGTAAACCTGGAGATCAAATTCATGTTTC
CATCACTGGTGTCAGTGTTTGGCGAGAAATCAGCATGGCCAAATGACAAAGGCGTGAAGAATAC
ACTGATGTTTCATGATCTTTATGAACAAGGAGTCAATGACACTGAACAATCTCAAGATATTCAT
CAATATGGAACGGTGTGGAATCTTATTCCAGGGGTCATCAGTACTATGTATGAGCATCAGCGTG
AAGCATTTGAATTTATGTGGACAAATTTAGTTGGTGATATTAGACTTGATGAGATAAAGCATGG
AGCAAAACCTGATGTTGTTGGTGGATGTGTTATCTGTCATGCTCCTGGAACAGGAAAGACACGA
TTAGCTATTGTATTTATCCAGACATACATGAAAGTGTTTCCAGACTGTCGGCCAGTGATTATTG
CACCACGTGGTATGCTCTTTGCTTGGGATGAGGAATTTAAGAAATGGAATGTTGATGTTCCTTT
TCATATACTAAACACAACTGATTACACTGGAAAAGAGGACCGGGACATATGCAAGTTAATAAAG
AAAGAACATAGGACAGAAAAGTTGACAAGACTAGTCAAACTGCTTTCATGGAACAAAGGCCATG
GTATTCTTGGAATAAGTTATGGTCTGTACACAAAACTGACCTCTGAAAAACCTGGCTGCACAGA
AGAAAACAAAGTAAGAAGCATTCTTCTTGATAACCCTGGCTTACTTGTTCTTGATGAAGGACAT
ACACCTAGGAATGAGCGCAGTGTTATGTGGAAAACTCTAGGAAATGTGAAAACTGAGAAGCGTA
TAATTTTATCTGGAACTCCTTTTCAGAACAATTTTCTTGAGCTTTACAACATTCTTTGTCTGGT
AAGGCCTAGATTTGGTGAAATGTTTTTGACGAAGTCAAGAGTAGGTCGAAGACATTATGTCTCA
AAAAAGCAAAAGGATAAGTTTTCTGATAAATATGAAAAGGGTGTTTGGGCATCACTGACTAGCA
ATGTAACTGATGATAATGCGGAGAAAGTAAGATCAATATTGAAACCATTTGTTCATATACATAA
TGGCAATATTCTTCGAACTCTTCCAGGACTCAGGGAGAGTGTAATTATTTTGAAGCCTCTTCCC
CTTCAAAAGAGTATCATTAAAAAGGTGGAAAACATTGGTTCTGGTAACAACTTCGAACATGAAT
ATGTCATTTCTTTAGCTTCTACACACCCTTCCCTTGTAACCGCCATTAACATGTCTGAGGAGGA
AGCTTCACTTATTGATAAACCTATGCTTGCTAAAGTGAGATCAAATCCATATGAAGGGGTAAAA
ACAAGATTTGTGATCGAAGTTGTTCGTTTGTCTGAAGCATTAAGAGAGAAGGTTTTGATTTTTA
GCCAATTTATTCAGCCTCTAGAGTTGATTAAAGAGCATCTTCGCAAGTTCTTCAAATGGAGAGA
AGGGAAAGAAATTCTTCAAATGGATGGAAAGATCCTTCCAAGATATCGCCAGGCTTCCATTGAA
GCCTTCAATAATCCAAATAATGATTCCAGGGTGTTACTTGCATCTACAAGAGCATGCTGTGAAG
GGATTAGCTTGACAGGTGCTTCAAGAATTGTGCTTCTAGATGTTGTTTGGAACCCAGCTGTTGG
AAGGCAAGCCATCAGCAGAGCATTTAGGATAGGTCAGAAGAAATTTGTATATACATATAATTTG
ATAACTTATGGAACAGGTGAAGGTGACAAATATGATAGGCAAGCAGAAAAGGATCACTTATCCA
AGTTGGTCTTCTCTACAGAAGACGAGTTCAATAATGTTAGGAACATGTTATCTAAAGCTGAAAT
GGAGCACTGTTCTAAGTTTATCTCAGAAGATAAAGTTTTGGAGGAGATGACTTCCCACGATCAA
CTTAAAGGAATGTTTTTGAAGATCCATTATCCACCAACTGAGTCAAACATTGTCTATAGTTACA
ATCAAATTGCTACTGAGTGA

>rmr1-4 | coding sequence | 4308 bp

ATGGATCGCGCCACGCCGCGCGTTTGCGGCCGCCGCGGCGTATCCCAAGCGGCGGTGGAAGCTG
CGCCGTCCTCCTCCCGCGCGCGCCGCCGCGATAAGGCGCCGGCCGTTGTCATGGACCTTGGCGA
CGACGACTGCGGCGGTGGCGGGGCCAGGAAGACGGTTGGTGGCGCTGCAGGTAGGTGCGAGGGA
TCCACGAAGGCTCCTTCGCCTATGCTGCCGCCGATGATGGTGCCGGCGGGAGCGGTGGCGCTGC
GGACACGATCGAGGAGGCGGGCGATGCTGGCGGCGGCAGTGGTGGAAGAGGCGCCAACGAAGAA
GAAGAAGAAGGAAGGAGCGATCCCAGATGCCGCGGAGGCAACCCGTGGCCACGGGAGCAAGGCC
GCTGCGACCTCGATGGCGACGTCGAGCCATAAGCGGCGCGCTGGGACCTCGAGGTCGACGTCGA
GAGATAAGCGGCGCGCGCGCTCGGGACGTGCCTCGGAGCCAGCTCGCGTGGGCCGCGCGCGCAA
GCGCAAAAGGAACGAGTTGGAGGCCCCCGCTCGGAGAGAACGTGTGAAGGCGCCATGTGTCAGT
GAAAGTGATGACAACAGCGGGCGAGGCGATGACGCTTCTCATGACGGGGATGCGGAGCCTCGCG
GCGGGGTCGCCATTGGCACTGATCTGGTTAACGGGGACCATCCGGCAGCTAAAGGGGTAGTGGA
AGGTGCTGGTGACGAGGACACAGGGGACGGAGGGAACAGTGGCCTGGCATCGACTGCTGATGTG
GTTGCTGAGGAGATGGCACCCTTTGAAGATGACTACGATGATGAGATGTTGGAGGAGCAGCTTG
TTGGAGATGTGATTCGTGCTTACAGTAATGGCAGAAACTTAGATTCAGATGGAGTGGATTGGGA
GGCAGAGGATGAGATGGAGTTCAATGACGATGCTGACAATAGTGATTTTATGGATGATGCTGAC
GATAGTGATTTTATGGATGATGCTTATGAAGGTGGCAATTCCAAACCAATTCAAAATCATGCTA
AGTTGGAAATACAAGACTGGGTGAACCAGAAAGTTGTTTTGAGTGGAGGGAGGTGTGAGGTGAG
GGGCGAGGGGGATCTGGAGGAAGAATTGGGTGTGGGAAAGGAAGCAGATGAGGAGGACGTGGAA
CCAAAGAGTGAAGCAGCTCCAGGTTCTGATAAAAGGGTCTTGCAGTTAGAAATCCTAGGTTCCG
ATGAGGAAATCAAGGTGCTTGAAAATATGAGTAGTGCCCCATCCAGGAAGGCGTCAGTTCAATC
GAAGTTACCAACTATTCCATCTTGTGTTGCATGGAGAACCCGATCATCATGGGGGGTAAATCAA
GATAGACTATCGTACGATACATATTTTGAGGAATTATCTGATGAGCCAAAAGAGGATGATGATG
ATACAGAGGTGGAACTTGATGAAGTTGAGGATGACAACAATGACGATGACAGTAGTGATGCTTA
TGATAAAGATGACGAAGAGAAGGAGGAAGAGGAAGAAGAGGCTGAAAGAAGAAAACTTAATAAC
AGGATTTGCACATCCGATGAAGACATGATCAATATTACTGTTCCTACATCAAGATATGATATGT
TTAAGAAAAAAAATTCCTCAAGATATGATATTGAGTGGGTGGAGGATGAAGATGCAAGTGTTGA
TATGTTACAGCCAGTTTCCTTTAAGAAAGATAGCAGCTGGAAGCCTGTGGCTGTTGGCAACGAC
ACATTTACTGAGCAACAAAAGCGATCACGATTTACTTGGGAGCTTGAGAGGAGGAAAAAGCTTA
AGCTTGAGATGAAGACAAATCCTTTGCATGAGCGGGATTTGGACTCAGATCCGAACTCATCAGG
TTCTGACCAGATCAGAAAGTATGGTTTCAAAAGTGATGGGAGTCATAAAGTTGATAGGAAAAAG
AAGCATACATCGCCCAAATCGGGCAAGAAACCCAGCAGCGCAATCATACTAAAGCGGCAGTCTC
TTTTGAAGCTTTTGGTAGATAAAATGAGTGGTGATAAAAGTTTAGCATCTTTTCCATTTGATCA
GAATCCTCAGCTTCAGTTTATTTTCAAAGAAATGCATCCATTGGTATTTTCATTTGGAGATGAA
GATCTAGTAGCAGCTGACAGGCCAGAGCAAGATGTTGGATTGGATATGTTATGGGCTGACTTTG
ACTTTGCTTTAGAGTCTGAGAATATCGGTACTTATTATGATGATGAGTGTCAAGAAGGCAATCA
ACTAGATTTTTCTCTTGCCTCAGTAACACCCTGTTCTCGTGGGAAGCATGAATTTGTTATTGAT
GATCAAATAGGGATCAGATGCAAATACTGTTCGTTGGTAAACCTGGAGATCAAATTCATGTTTC
CATCACTGGTGTCAGTGTTTGGCGAGAAATCAGCATGGCCAAATGACAAAGGCGTGAAGAATAC
ACTGATGTTTCATGATCTTTATGAACAAGGAGTCAATGACACTGAACAATCTCAAGATATTCAT
CAATATGGAACGGTGTGGAATCTTATTCCAGGGGTCATCAGTACTATGTATGAGCATCAGCGTG
AAGCATTTGAATTTATGTGGACAAATTTAGTTGGTGATATTAGACTTGATGAGATAAAGCATGG
AGCAAAACCTGATGTTGTTGGTGGATGTGTTATCTGTCATGCTCCTGGAACAGGAAAGACACGA
TTAGCTATTGTATTTATCCAGACATACATGAAAGTGTTTCCAGACTGTCGGCCAGTGATTATTG
CACCACGTGGTATGCTCTTTGCTTGGGATGAGGAATTTAAGAAATGGAATGTTGATGTTCCTTT
TCATATACTAAACACAACTGATTACACTGGAAAAGAGGACCGGGACATATGCAAGTTAATAAAG
AAAGAACATAGGACAGAAAAGTTGACAAGACTAGTCAAACTGCTTTCATGGAACAAAGGCCATG

GTATTCTTGGAATAAGTTATGGTCTGTACACAAAACTGACCTCTGAAAAACCTGGCTGCACAGA
AGAAAACAAAGTAAGAAGCATTCTTCTTGATAACCCTGGCTTACTTGTTCTTGATGAAGGACAT
ACACCTAGGAATGAGCGCAGTGTTATGTGGAAAACTCTAGGAAATGTGAAAACTGAGAAGCGTA
TAATTTTATCTAGAACTCCTTTTCAGAACAATTTTCTTGAGCTTTACAACATTCTTTGTCTGGT
AAGGCCTAGATTTGGTGAAATGTTTTTGACGAAGTCAAGAGTAGGTCGAAGACATTATGTCTCA
AAAAAGCAAAAGGATAAGTTTTCTGATAAATATGAAAAGGGTGTTTGGGCATCACTGACTAGCA
ATGTAACTGATGATAATGCGGAGAAAGTAAGATCAATATTGAAACCATTTGTTCATATACATAA
TGGCAATATTCTTCGAACTCTTCCAGGACTCAGGGAGAGTGTAATTATTTTGAAGCCTCTTCCC
CTTCAAAAGAGTATCATTAAAAAGGTGGAAAACATTGGTTCTGGTAACAACTTCGAACATGAAT
ATGTCATTTCTTTAGCTTCTACACACCCTTCCCTTGTAACCGCCATTAACATGTCTGAGGAGGA
AGCTTCACTTATTGATAAACCTATGCTTGCTAAAGTGAGATCAAATCCATATGAAGGGGTAAAA
ACAAGATTTGTGATCGAAGTTGTTCGTTTGTCTGAAGCATTAAGAGAGAAGGTTTTGATTTTTA
GCCAATTTATTCAGCCTCTAGAGTTGATTAAAGAGCATCTTCGCAAGTTCTTCAAATGGAGAGA
AGGGAAAGAAATTCTTCAAATGGATGGAAAGATCCTTCCAAGATATCGCCAGGCTTCCATTGAA
GCCTTCAATAATCCAAATAATGATTCCAGGGTGTTACTTGCATCTACAAGAGCATGCTGTGAAG
GGATTAGCTTGACAGGTGCTTCAAGAATTGTGCTTCTAGATGTTGTTTGGAACCCAGCTGTTGG
AAGGCAAGCCATCAGCAGAGCATTTAGGATAGGTCAGAAGAAATTTGTATATACATATAATTTG
ATAACTTATGGAACAGGTGAAGGTGACAAATATGATAGGCAAGCAGAAAAGGATCACTTATCCA
AGTTGGTCTTCTCTACAGAAGACGAGTTCAATAATGTTAGGAACATGTTATCTAAAGCTGAAAT
GGAGCACTGTTCTAAGTTTATCTCAGAAGATAAAGTTTTGGAGGAGATGACTTCCCACGATCAA
CTTAAAGGAATGTTTTTGAAGATCCATTATCCACCAACTGAGTCAAACATTGTCTATAGTTACA
ATCAAATTGCTACTGAGTGA
