# Supplementary material for: A Novel Snf2 Protein Maintains trans-Generational Regulatory States Established by Paramutation in Maize
Source: PLoS Biol. 2007 Oct 16;5(10):e275. doi: 10.1371/journal.pbio.0050275 (PMC2020503; doi:10.1371/journal.pbio.0050275)
Supplement: Supplementary file 2 [file pbio.0050275.sd002.doc]

# Protocol S1

**Complementation tests of new mutant alleles.** Complementation test results (Table S3) show ems98292 and ems98941 mutations define alleles of *mop1* and that ems98287 and ems051069 mutations define alleles of the *rmr1* locus. The ems98287, ems98292, and ems98291 mutations were isolated using an ems pollen mutagenesis and genetic screening strategy as previously described [^[1]^]. The ems051069 mutation was similarly generated and isolated using color converted A619 and A632 inbred parental lines [^[2]^].

Homozygous ems98262 /ems98262 plants having a Pl-Rh-like phenotype (ACS 7) were outcrossed to *Pl′* / *Pl′* testers. All F1 progeny plants (39 individuals from 3 independent outcrosses) had a Pl′-like anther phenotype (5 ACS 1; 22 ACS 2; 12 ACS 3) indicating the ems98262 allele is recessive and further that *Pl′* alleles transmitted from homozygous ems98262 /ems98262 plants are capable of inciting paramutation. The Pl-Rh-like anther phenotype was recovered in 10/62 F2 plants derived from three independent self-pollinations of F1 plants (30 ACS 1; 15 ACS 2; 2 ACS 3; 10 ACS 7). The observed frequency of Pl-Rh-like phenotypes (16%) is not significantly different from the 25% expected from a single locus recessive mutation (Pearson’s goodness of fit χ2 = .28; *P* = .59). All F2 progeny with ACS 7 anthers were ~2/3 the height of their Pl′-like siblings. Five of the 62 F2 progeny produced barren (no flowers) tassels so it was not possible to assign these to a given Anther Color Score class. However, these five anther-less progeny were short in stature and had very strong plant color similar to all other F2 plants that had ACS 7 anthers. If we include these five anther-less progeny among the "ACS 7" mutant class, then 15/62 (24%) of the F2 progeny have the mutant phenotype. These F2 segregation ratios are consistent with the hypothesis that the dark-anther and dark-plant phenotypes are due to a single locus recessive mutation. Homozygous ems98941 /ems98941 plants having a Pl-Rh-like phenotype were outcrossed to *Pl′*/*Pl′* testers. All F1 progeny plants (14 individuals from 2 independent outcrosses) had a Pl′-like anther phenotype (2 ACS 1; 9 ACS 2; 2 ACS 3; 1 ACS 4) indicating that the ems98941 allele is recessive and further that *Pl′* alleles transmitted from homozygous ems98941 /ems98941 plants are not recalcitrant to subsequent paramutation.

Results of genetic crosses (Table S3) indicate the ems98262 and ems98941 mutations complement mutations at *rmr1*, *rmr2* and *rmr6*. However, the two mutations fail to complement each other and the *mop1-1* mutation. If the ems98262 mutation represents an allele of *mop1*, then 1/2 of all progeny (14-15 individuals) from the two complementation crosses with *mop1-1* should have a Pl-Rh-like anther phenotype. The observed frequency of Pl-Rh-like types is not significantly different from the expected frequency for this hypothesis (Pearson’s goodness of fit χ2 = 3.07; *P* = .08) suggesting ems98262 defines an allele of *mop1* hereafter designated *mop1-3*. If the ems98941 mutation defines an allele of *mop1*, then 50% of the complementation cross progeny made with *mop1-1* (9-10 individuals) should have a Pl-Rh-like anther phenotype. The observed frequency is not significantly different from this hypothesis (Pearson’s goodness of fit χ2 = .67; *P* = .41) suggesting ems98941 defines an allele of *mop1* hereafter designated *mop1-4*. These assignments are consistent with the complementation results of *mop1-3* heterozygotes crossed by *mop1-4* homozygotes (Table S3). If the two mutations represent alleles of *mop1*, then 1/2 of all progeny (13 individuals) from the two complementation crosses should have a Pl-Rh-like anther phenotype. The observed frequency of Pl-Rh-like types is not significantly different from the expected frequency for this hypothesis (Pearson’s goodness of fit χ2 = 2; *P* = .16).

Plants homozygous for the ems98287 mutation, having a Pl-Rh-like phenotype, were outcrossed to *Pl-Rh* / *Pl-Rh* plants. Almost 1/2 of F1 plants (8 of 25 individuals from two independent outcrosses) had a Pl-Rh anther phenotype (2 ACS 1; 12 ACS 2; 2 ACS 4; 1 ACS 5; 8 ACS 7) suggesting ems98287 might represent a dominant mutation. However, the Pl-Rh-like phenotype was recovered in 12/40 (30%) F2 plants derived from two independent self-pollinations of ACS 3 F1 plants (3 ACS 1; 19 ACS 2; 5 ACS 3; 1 ACS 4; 12 ACS 7). The observed frequency of F2 mutant phenotypes (30%) is not significantly different from the 25% expected from a single locus recessive mutation (Pearson’s goodness of fit χ2 = .25; *P* = .62) and is consistent with the hypothesis that the dark-anther phenotype, is due to a single locus recessive mutation. Complementation test results (Table S3) indicate the ems98287 mutation complements mutations at *rmr2*, *rmr6*, and *mop1*. If the ems98287 mutation was an allele of *rmr1*, then 1/2 of all progeny (24-25 individuals) from the two complementation crosses should have a Pl-Rh-like anther phenotype. The observed frequency of Pl-Rh-like types (20/49) is not significantly different from the expected frequency for this hypothesis (Pearson’s goodness of fit χ2 = .83; *P* = .36), suggesting the ems98287and *rmr1-1* mutations define the same locus. Subsequent molecular mapping of the ems98287 mutation also placed it in the same position as *rmr1-1* (bin6.05) thus allowing us to conclude that ems98287 defines an allele of *rmr1* hereafter designated *rmr1-3*.

Using color-converted A619 and A632 inbred lines[2], EMS pollen mutagenesis [1] was repeated and M2 families were screened for the appearance of plants with darkly colored anthers. In separate growouts, the M2 progeny designated 34833 segregated 5/27 plants with ACS 7 anthers. This frequency is not statistically different from the 25% expectation for a single locus recessive mutation (Pearson’s goodness of fit χ2 = .33; *P* = .56) plants with ACS 7 anthers. Pollen from a single ACS 7 plant was used for genetic complementation crosses. This mutation (ems051069) fully complemented mutations defining *mop1*, *rmr2* and *rmr6* but failed to complement both *rmr1-1* and *rmr1-3* (Table S3). If the ems051069 mutation represents an allele of *rmr1*, then 1/2 of all progeny (26-27 individuals) from the two *rmr1* complementation crosses should have a Pl-Rh-like anther phenotype. The observed frequency of Pl-Rh-like types (28/53) is not significantly different from the expected frequency for this hypothesis (Pearson’s goodness of fit χ2 = .08; *P* = .77), suggesting the ems051069 mutation defines the same locus. The allele defined by the ems051069 mutation is hereafter referred to as *rmr1-4*.

***Pl′* establishment tests.**  Table S1 details crosses done to generate families for genetic tests used to determine the effect of *rmr1* mutations on establishment of *Pl′* states. Families 05-541 and 05-542 were segregating *Pl′ rmr1-1 / T6-9 Pl-Rh rmr1-2* plants (Figure 4)*.* These plants were selected using the dark anther mutant phenotype and then crossed to or by a *Pl-Rh* homozygous tester. Plants in families 05-103 and 05-539 are the color-converted A632 and A619 testers respectively. The structural genotype (presence or absence of the *T6-9* interchange pair) was scored in the field by assaying pollen fertility. The state of the *Pl1-Rhoades* allele was assayed by evaluating anther pigmentation on a 1 to 7 scale as previously described [^[3]^]. Plants with an ACS of 1-4 (representing the *Pl′* / *Pl′* phenotype) are referred to as having light anthers , plants with an ACS of 7 (representing the *Pl-Rh* / *Pl-Rh* phenotype) are referred to as having dark anthers, and plants with an ACS of 5-6 phenotype are described as intermediate (Figure 5).

***B′* establishment tests.** To test the role of *rmr1* in *b1* paramutation, isogenic *B-I* and *B′* stocks (*B1-I* ; *Pl1-Rhoades*) were first pollinated by *rmr1-1* / *rmr1-1* ; *b1-W23* / *b1-W23*; *Pl1-Rhoades / Pl1-Rhoades* plants. Intercrossing the resulting F1 plants (*B-I* / *b1-W23*  *B′* / *b1-W23*) gave rise to progenies in which there was segregation of Pl-Rh-like and Pl′-like plants. Among these Pl-Rh-like plants, the following *b1* genotypes occurred at a 1:1:1:1 frequency: *b1-W23* / *b1-W23* ; *b1-W23* / *B′* ; *b1-W23* / *B-I* ; *B-I* / *B′*. Blind test crosses of parental plants displaying dark plant colors (*rmr1-1* / *rmr1-1* ) were made to *b1-CO159* / *b1-CO159* pistillate testers (*Pl-Rh* / *Pl-Rh* ; CO159 / W23 stock). Actual *b1* genotypes of the staminate parents were assigned *ex post facto* based on segregation of plant color types among resulting test cross progenies (Table S2). An identical strategy using *rmr6* mutations has been described [2].

***In vitro* transcription reactions.** This (Figure S1) was carried out as described [^[4]^] with husk nuclei isolated from single ears of the same genetic stocks used to measure *pl1* RNA differences in anthers [1]. The anthocyanin genotype of these plants is *B1-I* (*B-I*)/*B1-I* (*B-I*); *Pl1-Rhoades* (*Pl′*) *Rmr1*/*Pl1-Rhoades* (*Pl′*) *rmr1-1* and *B1-I* (*B-I*)/*B1-I* (*B-I*); *Pl1-Rhoades* (*Pl′*) *rmr1-1*/*Pl1-Rhoades* (*Pl′*) *rmr1-1*. The same procedure was applied to single ears of *B1-I* (*B-I*)/*B1-I* (*B-I*); *Pl1-Rhoades* (*Pl′*) *Rmr1*/*Pl1-Rhoades* (*Pl′*) *rmr1-3* and *B1-I* (*B-I*)/*B1-I* (*B-I*); *Pl1-Rhoades* (*Pl′*) *rmr1-3*/*Pl1-Rhoades* (*Pl′*) *rmr1-3* plants with the following results: four biological replicates *pl1*, 1.4+/- 0.4; *b1*, 1.3+/- 0.6 ; *a1*, 6.2+/- 1.2 (mean difference +/- s.e.m.). Additional details regarding stock syntheses are available on request.

**Alignment of the helicase domain of RMR1 with other known and predicted Snf2 protein family members.** MUSCLE [^[5]^] was used to generate the alignment of the Snf2 helicase domains. The alignment figure (Figure S2) was shaded with GeneDoc 2.6.03 using a combination of manual and identity modes, with RMR1 being selected as the identity comparison sequence. The lightest level of shading represents conserved residues in at least 60% of the sequences, the middle level is conserved residues is at least 80% of the sequences, and the darkest level is amino acid identity in all sequences.

**RT-PCR expression analysis**. Figure S5. Fully expanded adult leaf (fourth node), flag leaf (terminal leaf), immature tassel, and immature ear tissue was harvested from an adult B73 elite inbred plant 83 days post-germination. Seedling leaf tissue was harvested from a non-mutant seedling from the F2 *rmr1-1* mapping population 10 days post-germination. Seedling root tissue was harvested from a non-mutant seedling from the F2 *rmr1-3* mapping population 10 days post-germination. Shoot apical meristem tissue was collected from a seedling(color converted A619 stock) 10 days post-germination. RNA was isolated from the above tissues using TRIzol reagent (Invitrogen, Carlsbad, California) and following the manufacturer’s protocol. 10 μg of total RNA was treated with DNase I (Invitrogen) and then reverse transcribed using SuperScript II reverse transcriptase (Invitrogen) and Oligo dT_(17)_ primers (Promega). The resulting cDNA was PCR amplified using *rmr1* specific primers Os11F and Os15R (Table S4). RT-PCR products sized on a 1% agarose gel and stained with ethidium bromide for visualization. Amplifications of *rmr1* from cDNA yields a 640 bp product, while amplification of *rmr1* from genomic DNA generates a 2663 bp product (not shown). RT-PCR was also carried on the cDNA using primers specific to *alanine aminotransferase* (*aat*) to ensure equal starting amounts of RNA [^[6]^]. Amplification of *aat* cDNA generates a 281 bp product and amplification from genomic DNA produces a 454 bp product. The DNA control was isolated from a color-converted A632 plant as described previously [^[7]^].

**Genetic mapping of *rmr1***. The CAPS marker for *rmr1-1* was identified by PCR amplifying genomic DNA from the mapping population with primers 1CAPS6F and 1CAPS6R (Table S4) and digesting the product with *Pvu*II (NEB, Ipswich, MA). Digested samples represent individuals with the non-mutant polymorphism. For the *rmr1-3* population, (134 individuals, 268 chromosomes scored) the primers were 1CAPS10F and 1CAPS10R, the restriction enzyme was *Bcl*I (NEB, Ipswich, MA), and digested samples represent mutant individuals. No recombinants were found with either marker.

**Candidate gene selection and sequencing**. See Table S4 for all oligonucleotide primer sequences used. A BLAST search using the rice *Os05g32610* ORF as a query identified maize GSS sequences CG886593, BZ668661, BZ681915, CZ392826, BH878936, CG882444 and sorghum EST sequences AW287235, BG322766, AW285838, BG323020. Sequence was generated for *rmr1* from PCR amplicons from various primer pairs. The Os10F / Os10R primer pair amplified sequence identifying the *rmr1-3* lesion, and the exonF / exonR amplicons contain the *rmr1-1, rmr1-2* and *rmr1-4* lesions. To verify the intron/exon structure of cDNA amplified across intron 1 with primers Intron0 F2/Intron0 R2, and across introns 2 and 3 with primers Os11F/Os15R. Additional sequence information was provided by the following primers: mid5′F, Os12F, Os13Rb, intronF, and intronR.

Small RNA northern blots. The blots were shown in Figure S6 were carried out as described in the Materials and Methods of the main text with the exception that the DNA oligonucleotides used did not contain *doppia* sequence, meaning the blots had to be striped and rehybridized with the complementary oligos end-labeled with polynucleotide kinase (Fermentas, EK0031) for accurate sizing.

1. . Hollick JB, Chandler VL. (2001) Genetic factors required to maintain repression of a paramutagenic maize *pl1* allele. Genetics 157(1): 369-378.
2. . Hollick JB, Kermicle JL, Parkinson SE. (2005) *Rmr6* maintains meiotic inheritance of paramutant states in *Zea mays*. Genetics 171(2): 725-740.
3. . Hollick JB, Patterson GI, Coe EH,Jr, Cone KC, Chandler VL. (1995) Allelic interactions heritably alter the activity of a metastable maize *pl* allele. Genetics 141(2): 709-719.
4. . Hollick JB, Patterson GI, Asmundsson IM, Chandler VL. (2000) Paramutation alters regulatory control of the maize *pl* locus. Genetics 154(4): 1827-1838.
5. . Edgar RC. (2004) MUSCLE: Multiple sequence alignment with high accuracy and high throughput. Nucleic Acids Res 32(5): 1792-1797.
6. . Woodhouse MR, Freeling M, Lisch D. (2006) Initiation, establishment, and maintenance of heritable *MuDR* transposon silencing in maize are mediated by distinct factors. PLoS Biol 4(10).
7. . Dorweiler JE, Carey CC, Kubo KM, Hollick JB, Kermicle JL, et al. (2000) *Mediator of paramutation1* is required for establishment and maintenance of paramutation at multiple maize loci. Plant Cell 12(11): 2101-2118.
