# Supplementary material for: A Novel Snf2 Protein Maintains trans-Generational Regulatory States Established by Paramutation in Maize
Source: PLoS Biol. 2007 Oct 16;5(10):e275. doi: 10.1371/journal.pbio.0050275 (PMC2020503; doi:10.1371/journal.pbio.0050275)
Supplement: Supplementary file 3 [file pbio.0050275.st001.doc]

| Parental cross | *Pl-Rh* tester | Progeny structural genotype | No. of individual progeny with specific anther color scores | | | | | | |
| --- | --- | --- | --- | --- | --- | --- | --- | --- | --- |
|  |  |  | 1 | 2 | 3 | 4 | 5 | 6 | 7 |
|  | | | | | | | | | |
| 05-542-6 X 05-103-1 | A632 | + / + | 0 | 3 | 0 | 0 | 0 | 0 | 0 |
| 05-103-1 X 05-542-6 | A632 | + / + | 1 | 6 | 0 | 2 | 0 | 0 | 0 |
| 06-530 X 06-316-10 | A632 | + / + | 0 | 2 | 3 | 0 | 0 | 0 | 0 |
| 05-539-1 X 05-542-6 | A619 | + / + | 1 | 2 | 0 | 1 | 0 | 0 | 5 |
| 05-539-2 X 05-541-6 | A619 | + / + | 0 | 0 | 1 | 0 | 1 | 3 | 8 |
| 05-541-6 X 05-539-2 | A619 | + / + | 0 | 3 | 2 | 4 | 1 | 1 | 0 |
| 06-528 X 06-316-12 | A619 | *+ / +* | 0 | 0 | 0 | 3 | 2 | 5 | 4 |
| 06-528 X 06-316-10 | A619 | + / + | 0 | 0 | 0 | 0 | 0 | 0 | 11 |
| 06-528 X 06-316-10 | A619 | + / + | 0 | 0 | 0 | 0 | 0 | 1 | 7 |
|  | | | | | | | | | |
| 05-542-6 X 05-103-1 | A632 | *T / +* | 0 | 4 | 5 | 3 | 0 | 0 | 0 |
| 05-103-1 X 05-542-6 | A632 | *+ / T* | 0 | 5 | 0 | 2 | 0 | 0 | 1 |
| 06-530 X 06-316-10 | A632 | *+ / T* | 1 | 11 | 1 | 0 | 0 | 0 | 0 |
| 05-539-1 X 05-542-6 | A619 | *+ / T* | 0 | 0 | 1 | 0 | 1 | 0 | 5 |
| 05-539-2 X 05-541-6 | A619 | *+ / T* | 0 | 0 | 0 | 1 | 0 | 0 | 2 |
| 05-541-6 X 05-539-2 | A619 | *T / +* | 0 | 1 | 2 | 1 | 2 | 0 | 0 |
| 06-528 X 06-316-12 | A619 | *+ / T* | 0 | 4 | 2 | 1 | 0 | 0 | 0 |
| 06-528 X 06-316-10 | A619 | *+ / T* | 0 | 0 | 0 | 0 | 0 | 0 | 4 |
| 06-528 X 06-316-10 | A619 | *+ / T* | 0 | 0 | 0 | 0 | 0 | 0 | 5 |
|  | | | | | | | | | |
| Totals: |  | + / + | 2 | 16 | 6 | 10 | 4 | 10 | 35 |
|  |  | *T* / + | 0 | 5 | 7 | 4 | 2 | 0 | 0 |
|  |  | + / *T* | 1 | 20 | 4 | 4 | 1 | 0 | 17 |
