# Supplementary material for: A Novel Snf2 Protein Maintains trans-Generational Regulatory States Established by Paramutation in Maize
Source: PLoS Biol. 2007 Oct 16;5(10):e275. doi: 10.1371/journal.pbio.0050275 (PMC2020503; doi:10.1371/journal.pbio.0050275)
Supplement: Supplementary file 4 [file pbio.0050275.st002.doc]

| Staminate  parent | Parental plant phenotype | Allele tested | No. of progeny with specific plant phenotypes | |
| --- | --- | --- | --- | --- |
|  |  |  | B-I | B′ |
|  | | | | |
| 02-508-22 | Dark B-I | *rmr1-1* | 0 | 60 |
| 02-508-60 | B-I | *rmr1-1* | 4 | 30 |
| 02-514-8 | Dark B′ | *rmr1-3* | 0 | 53 |
| 02-514-73 | B-I | *rmr1-3* | 0 | 59 |
|  |  |  |  |  |
| Totals |  |  | 4 | 202 |
