# Supplementary material for: A Novel Snf2 Protein Maintains trans-Generational Regulatory States Established by Paramutation in Maize
Source: PLoS Biol. 2007 Oct 16;5(10):e275. doi: 10.1371/journal.pbio.0050275 (PMC2020503; doi:10.1371/journal.pbio.0050275)
Supplement: Supplementary file 5 [file pbio.0050275.st003.doc]

| Parents | | Progeny | | | | | | | |
| --- | --- | --- | --- | --- | --- | --- | --- | --- | --- |
| Pistillate | Staminate | No. of ears | No. of individuals with specific anther color scores | | | | | | |
|  |  |  | 1 | 2 | 3 | 4 | 5 | 6 | 7 |
| *mop1-3* (ems98262) | | | | | | | | | |
| + / *mop1-1* | ems98262 / ems98262 | 1 | 2 | 8 | 2 | 0 | 0 | 0 | 3 |
| ems98262 / ems98262 | + / *mop1-1* | 1 | 6 | 0 | 3 | 0 | 0 | 0 | 5 |
| + / ems98262 | *rmr1-1* / *rmr1-1* | 2 | 4 | 19 | 17 | 0 | 0 | 0 | 0 |
| + / ems98262 | *rmr2-1* / *rmr2-1* | 1 | 2 | 14 | 10 | 0 | 0 | 0 | 0 |
| + / ems98262 | *rmr6-1* / *rmr6-1* | 1 | 0 | 6 | 17 | 2 | 0 | 0 | 0 |
| + / *rmr6-1* | ems98262 / ems98262 | 1 | 6 | 10 | 7 | 1 | 0 | 0 | 0 |
| *mop1-4* (ems98941) | | | | | | | | | |
| + / *mop1-1* | ems98941 / ems98941 | 2 | 5 | 1 | 1 | 0 | 0 | 0 | 12 |
| + / *mop1-3* | ems98941 / ems98941 | 2 | 1 | 14 | 1 | 2 | 0 | 0 | 8 |
| *rmr1-1* / *rmr1-1* | ems98941 / ems98941 | 2 | 1 | 12 | 1 | 1 | 0 | 0 | 0 |
| *rmr2-1* / *rmr2-1* | ems98941 / ems98941 | 2 | 10 | 11 | 2 | 4 | 0 | 0 | 0 |
| + / *rmr6-1* | ems98941 / ems98941 | 2 | 7 | 15 | 2 | 1 | 0 | 0 | 0 |
| *rmr1-3* (ems98287) | | | | | | | | | |
| + / *mop1-3* | ems98287 / ems98287 | 1 | 10 | 7 | 4 | 0 | 0 | 0 | 0 |
| ems98287 / ems98287 | + / *rmr1-1* | 2 | 11 | 16 | 2 | 0 | 0 | 0 | 20 |
| + / ems98287 | *rmr2-1* / *rmr2-1* | 1 | 13 | 13 | 0 | 0 | 0 | 0 | 0 |
| ems98287 / ems98287 | *rmr6-1* / *rmr6-1* | 1 | 18 | 3 | 0 | 0 | 0 | 0 | 0 |
| *rmr1-4* (ems051069) | | | | | | | | | |
| + / *mop1-4* | ems051069 / ems051069 | 1 | 7 | 13 | 2 | 3 | 0 | 0 | 0 |
| + / *rmr1-1* | ems051069 / ems051069 | 1 | 0 | 7 | 6 | 1 | 0 | 0 | 13 |
| + / *rmr1-3* | ems051069 / ems051069 | 1 | 3 | 6 | 2 | 0 | 0 | 0 | 15 |
| + / *rmr2-1* | ems051069 / ems051069 | 1 | 8 | 17 | 4 | 0 | 0 | 0 | 0 |
| + / *rmr6-1* | ems051069 / ems051069 | 1 | 2 | 13 | 12 | 0 | 0 | 0 | 0 |
