# Supplementary material for: A Novel Snf2 Protein Maintains trans-Generational Regulatory States Established by Paramutation in Maize
Source: PLoS Biol. 2007 Oct 16;5(10):e275. doi: 10.1371/journal.pbio.0050275 (PMC2020503; doi:10.1371/journal.pbio.0050275)
Supplement: Supplementary file 6 [file pbio.0050275.st004.doc]

| Primer Name | Sequence (5′ -> 3′) |
| --- | --- |
| Os10F | GAA GAG TTG GGT GTG GGA AA |
| Os10R | AAC ATC TTG CTC TGG CCT GT |
| exonF | GTG TCA GTG TTT GCC GAG AA |
| exonR | CTA TAG TTA CAA TCA AAT TGC TAC TGA G |
| mid5′F | GTT GCA GCA ACA GAA TCT AGC |
| Os11F | AAG CAT ACA TCG CCC AAA TC |
| Os15R | CAC GCT GAT GCT CAT ACA TA |
| Os12F | CCC AAT CAT GCA ACT CCT CT |
| Os13Rb | CCA TAA CTT ATT CCA AGA ATA CC |
| IntronF | GTC GTT TCC CAG AAT AGA GTA ACT TTG |
| IntronR | GAT ATG CAG AAT ACT ACA TAT GTT TGA G |
| CAPS6F | GCA TCT TCG CAA GTT CTT CA |
| CAPS6R | TCG TGG GAA GTC ATC TCC TC |
| CAPS10F | ATG AGT AGT GCC CCA TCC AG |
| CAPS10R | TCA GCC TCT TCT TCC TCT TCC |
| Intron0 F2 | GAT GAC GCT TCT CAT GAC G |
| Intron0 R2 | ATC ATC GTA GTC ATC TTC AAA GG |
